# LPLAT10/LPEAT2 produces atypical phospholipids with an unsaturated FA at the *sn*-1 position

**DOI:** 10.1016/j.jlr.2026.101007

**Published:** 2026-02-23

**Authors:** Hiroki Kawana, Rikuta Kataoka, Yukitaka Sato, Hirofumi Onishi, Taiga Iwama, Yuta Shimanaka, Daisuke Saigusa, Kuniyuki Kano, Nozomu Kono, Junken Aoki

**Affiliations:** 1 Graduate School of Pharmaceutical Sciences, The University of Tokyo, Bunkyo-Ku, Tokyo, Japan; 2 Graduate School of Science and Technology, Nara Institute of Science and Technology, Nara, Japan; 3 Graduate School of Pharmaceutical Sciences, Tohoku University, Sendai, Miyagi, Japan; 4 Faculty of Pharmaceutical Sciences, Teikyo University, Itabashi-Ku, Tokyo, Japan

**Keywords:** LPLAT10, LPEAT2, LPCAT4, lysophospholipids, Land’s cycle, unsaturated FA, *sn*-1 position

## Abstract

Major membrane phospholipids (PLs) contain saturated FAs, such as palmitic acid (C16:0) and stearic acid (C18:0), at the *sn*-1 position. Although atypical PLs containing unsaturated FAs at the *sn*-1 position exist as minor components, the biosynthetic pathway responsible for their production has remained elusive. Here, we report that LPLAT10 (also known as LPEAT2 or LPCAT4) is a lysophospholipid acyltransferase responsible for generating PLs with an unsaturated FA at the *sn*-1 position. In vitro, LPLAT10 incorporated both saturated and unsaturated FAs into lysophosphatidylcholine, lysophosphatidylethanolamine, and lysophosphatidylserine, selectively at the *sn*-1 position. LPLAT10 appeared to have a relatively higher affinity for unsaturated FA-CoAs. Consistently, only PLs with unsaturated FAs such as oleic acid (C18:1), linoleic acid (C18:2), arachidonic acid (C20:4), and DHA (C22:6) at the *sn*-1 position decreased in the brain from *Lplat10*-deficient mice. Despite their low abundance, these atypical PLs may have specific roles, given that LPLAT10 is highly expressed in neurons and its encoding genes are highly conserved among vertebrates above fish.

Typical phospholipid (PL) molecules in the cell have two asymmetric FAs attached to the two positions of their glycerol backbone; a saturated FA is found at the *sn*-1 position, whereas an unsaturated FA is at the *sn*-2 (1). This asymmetry of the two FAs in the PL molecules is widely conserved across species. Recent technological advances have enabled to analyze the binding positions of each FA in the glycerol backbone (*sn*-1 and *sn*-2) (2, 3, 4, 5). Using these techniques, it has been found that atypical PL species, with polyunsaturated FAs at the *sn*-1 position, which do not follow the fatty-acid asymmetry principle described above, also exist as minor components (2, 3, 4, 5). However, the molecular mechanisms producing atypical PL species, as well as their biological significance, have been poorly understood.

It has been believed that both the asymmetry and the FA composition of membrane PLs are thought to be determined by the PL remodeling reaction, also known as Lands’ cycle, in which FAs of newly biosynthesized PLs are replaced (1, 6). PL remodeling consists of two-step reactions: removing an FA from PL molecules and reintroducing a new FA into the resulting lysophospholipids (lysoPLs) in the first reaction. The enzymes that catalyze the first-step reaction are still unknown; however, the second step, the FA transfer reaction, is known to be catalyzed by lysophospholipid acyltransferases (LPLATs) (6, 7).

To date, 14 LPLATs (LPLAT1–14) have been identified and characterized. Structurally, they are classified into either the AGPAT or MBOAT families (6, 7). We have recently proposed a uniform LPLAT enzyme nomenclature (LPLAT1–14), as ambiguity and confusion were caused by multiple names being used to refer to the same enzyme or the same name being assigned to completely different enzymes (6). Here, we use the updated LPLAT nomenclature. Among these 14, LPLAT1–4 incorporate a FA into lysophosphatidic acid (LPA). Thus, they are thought to be LPA acyltransferases and are involved in PL synthesis in the de novo (Kennedy) pathway. Among the 10 LPLATs (LPLAT5–14) involved in PL remodeling, LPLAT12 (LPCAT3) and LPLAT11 (LPIAT1) have been the most extensively analyzed. LPLAT12 is an enzyme that mainly introduces arachidonic acid at the *sn*-2 position of phosphatidylcholine (PC) (8, 9, 10, 11, 12), whereas LPLAT11 introduces arachidonic acid at the *sn*-2 position of phosphatidylinositol (PI) (13, 14, 15). After the discovery of these LPLATs, the specific functions of arachidonic acid-containing PLs have been revealed through analyses using KO mice and SNPs in humans (14, 16, 17).

PL remodeling occurs both at the *sn*-2 and *sn*-1 positions (18, 19). The enzymes involved in the *sn*-1 remodeling, however, had not been characterized fully. One reason for this was the difficulty in preparing *sn*-2 lysoPLs (with an FA at the *sn*-2 position), which are used to assess FA incorporation into the *sn*-1 position (20). In general, *sn*-2 lysoPLs are unstable because the FA at the *sn*-2 position easily migrates to the *sn*-1 position by an intramolecular reaction, resulting in conversion to *sn*-1 type lysoPLs (20, 21). Previously, we found that this intramolecular FA transfer reaction is pH dependent and that we could completely suppress the reaction by adjusting the pH of the organic solvent to acidic (pH 4.0) (21). Accordingly, we have been able to prepare *sn*-2 lysoPLs in large quantities and evaluate FA incorporation into the *sn*-1 position (22, 23).

Using this novel approach, we found that the *sn*-1 LPLAT activities for major fatty acyl-CoA species, palmitoyl-CoAs and stearoyl-CoAs, were distributed in various tissues and cells (23). Furthermore, using membrane fractions from cells with knockdown of 14 different LPLATs as enzyme sources, we successfully identified two LPLATs that introduce palmitic acid (C16:0) and stearic acid (C18:0) at the *sn*-1 position (22, 23). First, LPLAT8 (LPCAT1) was found to have an activity of introducing palmitic acid into the *sn*-1 position of PC (22). Interestingly, LPLAT8 inserts palmitic acid at both the *sn*-1 and *sn*-2 positions (22). LPLAT8 was known to contribute to dipalmitoyl PC production in lung surfactants (24). The activity of LPLAT8 in introducing palmitic acid at both positions explains why LPLAT8 is a dipalmitoyl PC-producing enzyme. We and others recently identified another *sn*-1 LPLAT, LPLAT7 (LPGAT1), which incorporates stearic acid at the *sn*-1 position in PC and phosphatidylethanolamine (PE) (23, 25, 26). In LPLAT7 KO mice, the level of stearic acid at *the sn*-1 position in PLs was dramatically reduced in various tissues, with a compensatory increase in the level of palmitic acid at *the sn*-1 position (23, 25). LPLAT7 KO mice and zebrafish showed abnormalities in various tissues, including the muscle, liver, and testis (26, 27, 28).

In this study, as a part of our continuing studies to identify novel *sn*-1 LPLATs, we searched for LPLATs that introduce oleic acid (C18:1) at the *sn*-1 position. Consequently, we identified LPLAT10/LPEAT2/LPCAT4 as a candidate. Here, we report the biochemical characterization of LPLAT10, including an analysis of its endogenous substrates and products using KO mice.

## Materials and methods

### Reagents

FBS, penicillin-streptomycin-glutamine, Opti-MEM, Lipofectamine RNAiMAX, and Lipofectamine 2000 were obtained from Thermo Fisher Scientific (Waltham, MA). DMEM was purchased from Nissui Pharmaceutical (Tokyo, Japan). [^13^C_16_] C16:0-CoA and [^13^C_18_] C18:1-CoA were puchased from Taiyo Nippon Sanso (Tokyo, Japan). All glycerophospholipids, lysoPLs, and nonlabeled acyl-CoAs were purchased from Avanti Polar Lipids (Alabaster, AL). LC-MS grade methanol and acetonitrile were purchased from Kanto Chemical (Tokyo, Japan). Chloroform, formic acid, carboxymethylcellulose, Tris (hydroxymethyl) aminomethane, sucrose, EDTA, and ammonium formate were purchased from Fujifilm-Wako (Osaka, Japan). Honeybee venom phospholipase A_2_ (PLA_2_), lipase from *Rhizomucor miehei* (intrinsic PLA_1_), and p-nitroaniline were obtained from Merck (Darmstadt, Germany).

### Plasmid and siRNAs

For the construction of an expression vector for LPLAT10, complementary DNA (cDNA) for human LPLAT10 (gene official symbol: LPCAT4, NCBI accession number: NM_153613) was amplified by PCR using PrimeSTAR GXL Polymerase (TAKARA BIO, Inc) and human embryonic kidney 293A (HEK293A) cell cDNA as a template. The PCR primers used were as follows.

Forward: 5′-ATGGACTACAAGGATGACGATGATAAGAGCCAGGGAAGTCCGGGGG-3′

Reverse: 5′-GGGCATTGGCCATCGATCTCGATCAGTCTCCCTTCTGCTTGGGTGC-3′

The amplified cDNA fragments were inserted into the pCAGGS vector (a gift from J. Miyazaki, Osaka University) with an N-terminal FLAG-tag. The nucleotide sequences of the prepared plasmids were checked by DNA sequencing (FASMAC).

For the LPLAT knockdown experiment, Silencer Select siRNAs were obtained from Thermo Fisher Scientific (Waltham, MA). The catalog numbers of siRNA used in this study are shown below.

Negative control #1 5′-UAACGACGCGACGACGUAAtt-3′ (4390843), LPLAT5/AGPAT5 (s30735 and s30736), LPLAT6/LCLAT1 (s48420 and s48421), LPLAT7/LPGAT1 (s19258 and s19259), LPLAT8/LPCAT1 (s36575 and s36576), LPLAT9/LPCAT2 (s29823 and s29824), LPLAT10/LPCAT4 (s48551 and s48552), LPLAT11/MBOAT7 (s35614 and s35616), LPLAT12/LPCAT3 (s19799 and s19800), LPLAT13/MBOAT2 (s43424 and s43425), and LPLAT14/MBOAT1 (s45847 and s45848).

### Cell culture and transfection

HEK293A cells (purchased from Thermo Fisher Scientific) were maintained in DMEM supplemented with 10% FBS and penicillin-streptomycin-glutamine at 37°C in the presence of 5% CO_2_ gas. Transfections were performed using OptiMEM and lipofection reagent (Lipofectamine 2000 for plasmid transfection and Lipofectamine RNAiMAX for siRNA transfection). Typically, on day 1, HEK293A cells were seeded in a culture plate or a dish. On day 2, transfections were performed according to the manufacturer's instructions. Cells were collected 24 h after the plasmid transfection and 48 h after the siRNA transfection for subsequent experiments. Lipofectamine is known to contain a significant amount of dioleoyl PE. Consequently, this may affect the measurement of PE species. Under the transfection conditions employed in this study, we have confirmed that the use of Lipofectamine did not alter the amount of PLs, including dioleoyl PE.

### Total RNA extraction, reverse transcription, and quantitative PCR

Total RNA extraction from HEK293A cells was performed using the GenElute™ Mammalian Total RNA Miniprep Kit (SIGMA) according to the manufacturer's protocol. To eliminate genomic DNA contamination, DNase treatment was performed on the extracted total RNA before quantitative PCR (qPCR). The sample was mixed with reaction buffer and DNase I (SIGMA), incubated at 37°C for 30 min, then stop buffer was added, and the mixture was incubated at 70°C for 10 min. Reverse transcription was performed using random hexamer primers and the High-Capacity cDNA RT Kit (Applied Biosystems). Reactions were performed using a PCR thermal cycler (Takara Bio) at 25°C for 10 min, 37°C for 120 min, and 85°C for 5 min. Samples were stored at −20°C after the reaction. qPCR was performed under the following conditions. Primers were designed using Primer-BLAST to span an exon-exon junction, amplifying a region of 80 bp to 150 bp. The primer sequences for each LPLAT are shown below. Reactions were carried out using prepared cDNA as a template, mixed with SYBR Premix Ex Taq (TaKaRa) and primers (200 nM) using an ABI7200 real-time PCR (Applied Biosystems). The expression of each LPLAT was normalized to GAPDH expression levels and displayed as a relative value with the negative control group set at 100%.

qRT-PCR primer

LPLAT5

Forward (5′-3′): gacgcaggaactccaatgtatc; reverse (5′-3′): gcagcaaatgcctgactagc

LPLAT6

Forward (5′-3′): tcatgctgagtccctttttacc; reverse (5′-3′): ccaataatgccacaggtaggg

LPLAT7

Forward (5′-3′): acagtgatggaatggggagaag; reverse (5′-3′): tcatctgagcaacaaccagtc

LPLAT8

Forward (5′-3′): ctgtggaggaaggttgtggac; reverse (5′-3′): aagtaggacgagtgaggcg

LPLAT9

Forward (5′-3′): agtcctcctcagatacccaaac; reverse (5′-3′): gaactggcataaactcaacttctac

LPLAT10

Forward (5′-3′): atcgtcctctttctcctctggc; reverse (5′-3′): gcacacagtcttcctccatcctg

LPLAT11

Forward (5′-3′): tggctctggcctggactttctc; reverse (5′-3′): tcaccagcttcagcgtcagcag

LPLAT12

Forward (5′-3′): ctatgacaaccaccccttctg; reverse (5′-3′): atactccttctgtgaccagcc

LPLAT13

Forward (5′-3′): cccttttgggcctttatcttgc; reverse (5′-3′): agcagtaattgtgcatgttctcc

LPLAT14

Forward (5′-3′): ctcccgaagatgcaggatgg; reverse (5′-3′): ggcataccacaaaattcacctgg

GAPDH

Forward (5′-3′): gccaaggtcatccatgacaact; reverse (5′-3′): gaggggccatccacagtctt

### Preparation of membrane fractions

HEK293A cells or minced mouse tissues (20–100 mg) were homogenized in ice-cold TSC buffer (20 mM Tris-HCl [pH 7.4], 300 mM sucrose, and cOmplete protease inhibitor cocktail [Roche, Mannheim, Germany]) using a probe sonicator (Microtec, Chiba, Japan). After the homogenates were centrifuged at 800 *g* for 10 min, the supernatants were further centrifuged at 100,000 *g* for 1 h. The resultant pellets were resuspended in TSE buffer (20 mM Tris-HCl [pH 7.4], 300 mM sucrose, and 1 mM EDTA). After determining the protein concentration using the BCA protein assay kit (Thermo Fisher Scientific), the resulting membrane fractions were stored at −80°C until the LPLAT assay.

### Preparation of *sn-*2-dominant and *sn-*1-dominant lysoPLs

We prepared *sn*-2-dominant lysophosphatidylcholine (LPC) with oleic acid (C18:1) and deuterium-labeled palmitic acid-*d*_31_ (C16:0-*d*_31_) from dioleoyl (diC18:1) PC and di-palmitoyl-*d*_31_ (diC16:0-*d*_31_) PC, respectively, by PLA_1_ reaction as described previously (22). Briefly, dioleoyl (diC18:1) PC and di-palmitoyl-*d*_31_ (diC16:0-*d*_31_) PC were subjected to PLA_1_ reaction using *Rhizomucor miehei* lipase, which has significant PLA_1_ activity, and the resulting *sn*-2 lysoPLs (*sn*-2 oleoyl LPC and *sn*-2 palmitoyl-*d*_31_ LPC) were purified using a Bond Elut C18 solid phase cartridge column (Agilent Technologies) to remove the remaining PL substrates and free FAs. To stop a migration reaction of the *sn*-2-acyl LPCs, we used acidic methanol as a solvent and stored the *sn*-2-dominant LPCs at −80°C until use. The *sn*-1-dominant C18:1 LPC and *sn*-1-dominant C16:0-*d*_31_ LPC were prepared from the corresponding *sn*-2-dominant LPC isomers using the spontaneous acyl-migration reaction as described previously (23). The concentration of each LPC species was determined by LC-MS/MS. Other *sn*-2-dominant species, lysophosphatidylethanolamine (LPE), lysophosphatidylserine (LysoPS), lysophosphatidylglycerol (LPG), and LPA were prepared similarly from corresponding diC18:1 PLs. In the case of lysophosphatidylinositol (LPI), *sn*-2-dominant LPI was produced from soy PI (predominantly containing 1-C16:0-2-C18:2-glycerophosphoinositol).

### LPLAT assay

LPLAT assays were performed as previously described (22, 23). Briefly, the LPL solution in a test tube was dried up in a glass tube. Then, assay mixtures containing 100 mM Tris-HCl (pH 7.4), 0.03% Tween-20, and acyl-CoAs were added to the tube. The reaction was initiated by adding a membrane fraction and maintained at 37°C for 10 min. Reactions were stopped by the addition of chloroform/methanol (v/v:1/2). After adding corresponding PL internal standards (ISs), lipids were extracted by the Bligh and Dyer method. After the organic solvents were dried using a centrifugal evaporator, the lipids were reconstituted in methanol. LC-MS/MS was used to measure the resulting PLs.

### Determination of the position into which FAs are incorporated

The position determination experiment was performed as previously described (22, 23). Briefly, the LPLAT assay was performed using C16:0-*d*_31_ LPC (*sn*-2-dominant) as the acceptor and [^13^C_16_] C16:0-CoA as the donor. The products were subjected to a PLA_2_ reaction, and LC-MS/MS analyzed the resulting LPCs. To remove C16:0-*d*_31_ LPC from the products, the reaction mixtures were applied to a Bond Elut C8 solid-phase cartridge column (Agilent Technologies) before the PLA_2_ treatment. The products ([^13^C_16_] C16:0-C16:0-*d*_31_-PC) were eluted with 5 mM ammonium formate in 99.5% (v/v) methanol from the cartridge column. The eluted samples were concentrated by evaporation and dissolved in 0.1% Triton X-100 and 100 mM Tris-HCl buffer (pH 8.9). The PLA_2_ reaction was performed by adding 0.1 unit of bee venom PLA_2_ and incubating it at 37°C for 10 min. The PLA_2_ reaction was stopped by adding acidic methanol (pH 4.0) containing an IS. The samples were then subjected to LC-MS/MS analysis to determine the concentrations of *sn*-1-C16:0-*d*_31_ LPC and *sn*-1-[^13^C_16_]C16:0 LPC. In the analysis of FA distribution in diacyl-PLs from mouse brain, the extracted diacyl PLs were subjected to similar PLA_2_ treatment and analyzed the resulting LPLs by LC-MS/MS.

### Sample preparation for LC-MS/MS lipid analysis of culture cells and mouse tissues

HEK293A cells were washed with OptiMEM and then incubated with ice-cold acidic methanol (pH 4) containing PL ISs. After 10 min of incubation at room temperature, the samples were collected, passed through a 0.2 μm pore size filter (4 mm inner diameter; YMC), and subjected to LC-MS/MS analysis. Minced mouse tissues (∼20 mg) were homogenized in ice-cold acid methanol using a micro smash homogenizer (TOMY, Tokyo, Japan) with zirconia beads (3,000 rpm and 4°C for 2 min). After centrifugation (21,500 *g*, 5 min), the supernatant was diluted with acidic methanol containing ISs. The resulting samples were filtered and subjected to LC-MS/MS analysis.

### LC-MS/MS analyses

LPLs or diacyl PLs were quantified as previously described (23). Briefly, the LC-MS/MS system consisted of a Vanquish HPLC (high-performance liquid chromatography) and a TSQ Altis Triple-Stage Quadrupole mass spectrometer (Thermo Fisher Scientific), equipped with a heated-electrospray ionization-II source. For HPLC, samples were separated on an L-column 2 (100 mm × 2 mm, 3 μm particle size; CERI) for LPL analysis and a reverse-phase column (Capcell Pak C8 UG120, 150 mm × 1.5 mm, 5 μm particle size; Osaka Soda) for diacyl PL analysis. LPLs and diacyl PLs were detected by selected reaction monitoring (SRM) in positive ion mode. At MS1, the *m/z* values of [M+H]^+^ ions for LPC, PC, PE, and phosphatidylserine and [M+NH_4_]^+^ ions for PI, phosphatidylglycerol, and phosphatidic acid were selected. At MS3, phosphocholine fragments (*m/z* = 184.1) were detected for LPC and PC. Diacylglycerol fragments were detected for PE, phosphatidylserine, PI, phosphatidylglycerol, and phosphatidic acid. SRM was performed in the negative ion mode to characterize the acyl-chain composition of PL species. At MS1, [M+HCOO]^–^ ions for PC and [M-H]^–^ ions for other PLs were selected. At MS3, the *m/z* values of the corresponding FA negative ions.

Separation and quantification of *sn*-1 and *sn*-2 FA positional isomers in PC (C38:6) were performed using a modified method based on previous publications (2). The sample was mixed for 30 s with 20 μl/protein (concentration) of methanol containing IS (100x SPLASH LIPIDOMIX Mass Spec Standard; Avanti Research). The sample was sonicated for 5 min and then centrifuged at 16,400 *g* for 10 min at 4°C. The supernatant (50 μl) was transferred to a sample tube and then diluted 10 times with methanol. The sample (2 μl) was subjected to LC-MS/MS. The system consisted of an Acquity™ Ultra Performance LC I-class (Waters Corp, Milford, UK) interfaced to a Waters Xevo TQ-XS MS/MS equipped with electrospray ionization. The SRM transitions of the precursor ion to the product ion at positive ion mode for the PC (C38:6) and PC (C15:0/18:1-*d*_*7*_) were *m/z* 806.7/184.0 and 753.6/184.0, respectively. The cone voltage and collision energy for the detection were 10 V and 22 eV, respectively. The other settings are as follows: 3.5 kV capillary voltage, 64 V cone voltage, 50 l/h cone gas (N_2_) flow rate, 600°C desolvation temperature, and 1,000 l/h desolvation gas flow. LC separation was performed using the dual-connected reverse-phase column (CAPCELL PAK INERT C18 ACR 3 μm, 1.5 × 150 mm) with a gradient elution using solvent A (1 mol/l ammonium formate/water/formic acid = 2/98/0.0096, v/v/v) and B (1 mol/l ammonium formate/water/acetonitrile/formic acid = 1/4/95/1.160, v/v/v/v) at 0.2 ml/min: 1 to 100% B from 2.5 to 7.0 min, 100% B from 7.0 to 25.0 min, 100 to 1% B from 25.0 to 25.1 min, and 1% B for 5.0 min. The oven temperature was 55°C. The data were collected using the MassLynx v4.2 software (Waters Corp), and the peak areas were calculated by Traverse MS (Reifycs, Inc, Tokyo, Japan) for further statistical analysis.

### Mice

Mice were housed in specific-pathogen-free barrier facilities at the University of Tokyo and used in accordance with protocols approved by the Animal Care and Use Committee of the University of Tokyo (approval number: A2024P008).

### Generation of LPLAT10 KO mice

*Lplat10* KO mice were generated by using the clustered regulatory interspaced palindromic repeats (CRISPR)-CRISPR-associated protein 9 (Cas9) genome editing system. The fertilized eggs from C57BL/6J mice were injected with single guide RNA and recombinant Cas9 protein and transferred into the oviducts of pseudo-pregnant female mice. Single guide RNA-targeting sequences, including protospacer adjacent motif sequence (underlined), were as follows. 5′-CCTTCGGATCCGTGTTCGGGGTC-3′ (the target site is the antisense strand). The above procedures were performed at the Genetic Experiment Center, Faculty of Medicine, Yamagata University. The germline transmission was confirmed by mating the born female mice with C57BL/6J male mice (purchased from Japan SLC, Hamamatsu, Japan). F1 generation mice were subjected to DNA sequencing and selected for frameshift mutations. We established three KO mouse lines having different mutations and named them MT1, MT3, and MT7, respectively. For genotyping the MT1 and MT3 lines, DNA fragments were amplified using a set of primers from genomic DNA, and the resulting DNA fragments were separated and visualized by agarose gel electrophoresis. For genotyping MT7, the amplified DNA fragments were mixed with those of the wild type, and then, the heteroduplex mobility assay was performed. The sequence of the primers was as follows.

For wild type

Forward primer: 5′-TGCTTGGCTTCCTTCGGATC-3′

Reverse primer: 5′-CTGTCCTCTCTACACTGGGAGC-3′

For MT1

Forward primer: 5′-CCCTCGTACAATCGCAAACG-3′

Reverse primer: 5′-CCCTTTGACCCCGAACACG-3′

For MT3

Forward primer: 5′-CTGCTTGGCTTTCCTGCTAC-3′

Reverse primer: 5′-TGGGAGCCAGTGTTTCCATG-3′

For MT7

Forward primer: 5′-GACGACCAGCGTGTTTTGAG-3′

Reverse primer: 5′-GAGACACAACCTTGGGCAGG-3′

Western blotting confirmed that LPLAT10 protein expression was absent in all three lines (Supplemental Fig. S3B). The MT3 line was mainly used for subsequent lipid analyses and functional assays described in this study.

### Western blotting

Tissue lysates (20 μg per lane) were separated by SDS-PAGE and transferred onto a PVDF membrane. After transfer, the membrane was blocked with 5% skim milk in Tris-buffered saline with Tween-20 for 30 min at room temperature. The membrane was incubated overnight at 4°C with a primary antibody against LPLAT10 (17905-1-AP; Proteintech, 1:1,000 dilution), calnexin (Abcam, ab22595, 1:4,000 dilution), and voltage-dependent anion channel (Abcam, ab15895, 1:1,000 dilution), followed by a 1 h incubation at room temperature with an HRP-conjugated secondary antibody. Protein bands were detected using ECL reagent and visualized with Amersham Imager 680 (GE Healthcare).

### In situ hybridization

The mouse brain was isolated and fixed with 4% paraformaldehyde in PBS at room temperature. After fixation, the tissue was dehydrated and embedded in paraffin. A 5-μm sectioned tissue sample on 3-(trimethoxysilyl)propyl methacrylate-coated glass (Matsunami Glass; Matsunami, Osaka, Japan) was used for RNA in situ hybridization. RNA in situ hybridization was performed using the RNAscope 2.5 HD RED assay according to the manufacturer's instructions.

### Sample preparation for functional MS imaging

For functional MS imaging (fMSI), mouse brains were obtained from 7- to 8-week-old male wild-type or LPLAT10 KO mice, perfused with PBS, and immediately freeze-embedded in 2% carboxymethylcellulose using liquid nitrogen. The brains were cryosectioned at a thickness of 10 μm and thaw-mounted onto 3-(trimethoxysilyl)propyl methacrylate-coated glass slides (Matsunami, Osaka, Japan). Sample preparation for fMSI was performed as previously described (29). Briefly, brain sections were sprayed with the reaction mixture containing 200 mM Tris-HCl, 0.03% SDS, 100 μM [^13^C_18_] oleoyl-CoA, and 200 μM *sn*-2-dominant C18:1 LPC using an automated sprayer (SunCollect, SunChrom, Frieddominantsdorf, Germany). A total of 23 reaction mixture layers was applied at the 15 μl/ml flow rate (z position, 10mm; line distance, 2 mm; speed 900 mm/min; air pressure, 0.3MPa). After spraying, the sections were immediately placed in a SunDigest (SunChrom, Frieddominantsdorf, Germany) incubator at 37℃ for 3 min. The enzymatic reaction was stopped by washing the sections with 50 mM ammonium formate. After washing with 50 mM ammonium formate, the matrix, p-nitroaniline, dissolved in 80% EtOH containing 150 mM ammonium formate, was applied to sections using SunCollect.

### MALDI-MSI analysis

MALDI-MSI analyses were performed using Fourier transform orbital trapping MS (QExactive, Thermo Fisher Scientific, San Jose, CA) equipped with a MALDI laser system (AP-SMALDI5, TransMIT, Giessen, Germany) in full-scan mode at a mass resolution of 140,000. The laser was manually focused. The laser power was set at a 27° attenuator setting to yield optimal results. Ion images were reconstructed using IMAGEREVEAL™ MS (Shimadzu, Kyoto, Japan) with a mass tolerance of ±5.5 ppm.

### Phylogenetic tree analysis

Amino acid sequences corresponding to LPLAT8, LPLAT9, and LPLAT10 from *Homo sapiens*, *Mus musculus*, *Danio rerio*, *Callorhinchus milli*, *Ciona intestinalis*, *Branchiostoma floridae*, *Petromyzon marinus*, *Strongylocentrotus purpuratus*, *Amblyraja radiata*, *Carcharodon carcharias*, *Oryzias latipes*, *Scleropages formosus*, and *Cyprinus carpio* were retrieved from the NCBI database. Multiple sequence alignment was performed using Clustal W implemented in MEGA11 with default parameters. Phylogenetic relationships were inferred using the neighbor-joining method with the Poisson correction model for amino acid substitutions. Gaps and missing data were treated by complete deletion. One thousand bootstrap replicates evaluated the robustness of the tree topology. All analyses and tree visualizations were conducted in MEGA11. The accession numbers of the sequences are listed below.

*Homo sapiens* LPLAT8: NP_079106.3, LPLAT9: NP_060309.2, LPLAT10: NP_705841.2

*Mus musculus* LPLAT8: NP_663351.3, LPLAT9A: NP_766602.1, LPLAT9B: NP_081875.1, LPLAT10: NP_997089.1

*Danio rerio* LPLAT8A: NP_001037806.2, LPLAT8B: XP_073781776.1, LPLAT9: NP_001018492.1, LPLAT10A: XP_003201747.2, LPLAT10B: NP_991122.2

*Callorhinchus milli* LPLAT8: XP_007901220.2, LPLAT9: XP_007887679.1

*Branchiostoma floridae* LPLAT9: XP_035678837.1

*Strongylocentrotus purpuratus* LPLAT9: XP_030831214.1

*Petromyzon marinus* LPLAT8B: XP_032813329.1, LPLAT8A: XP_032826988.1

*Ciona intestinalis* LPLAT9B: XP_018671723.1, LPLAT9A: XP_002131598.1

*Cyprinus carpio* LPLAT8B: XP_042596198.1, LPLAT8D: XP_042601307.1, LPLAT8A: XP_042632710.1, LPLAT8C: XP_042611440.1

*Oryzias latipes* LPLAT8: XP_004077809.1, LPLAT9: XP_023809405.1, LPLAT10: XP_004079999.1

*Scleropages formosus* LPLAT8A: XP_018582616.2, LPLAT8B: XP_018618227.1, LPLAT9: XP_018597704.2, LPLAT10A: XP_029112267.1, LPLAT8B: XP_018601495.2

*Amblyraja radiata* LPLAT8: XP_032904202.1, LPLAT9: XP_032892205.1

*Carcharodon carcharias* LPLAT8: XP_041039792.1, LPLAT9: XP_041046653.1

## Results

### LPLAT10 is a candidate *sn*-1 LPLAT for oleic acid

**Fig. 1:**
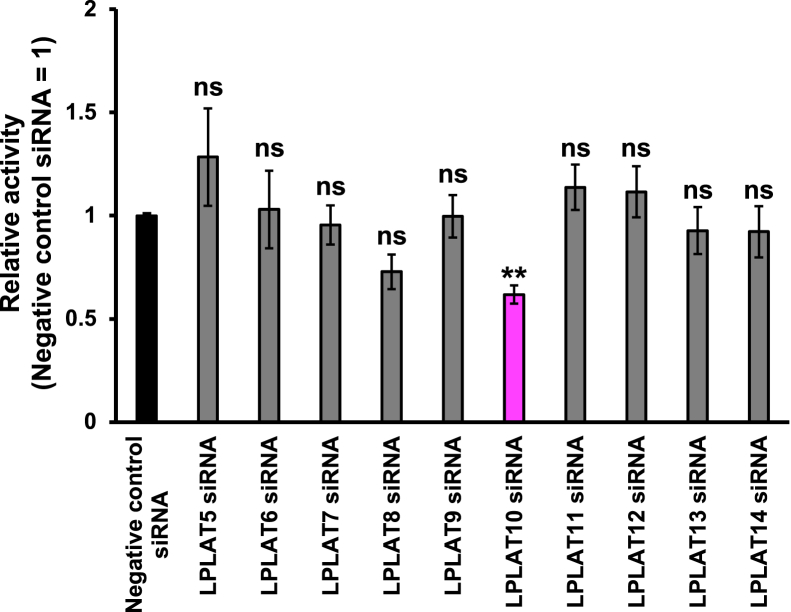
Screening of LPLATs responsible for oleic acid incorporation at the *sn*-1 position of PC expression of 10 LPLATs was suppressed in HEK293A cells using siRNAs, and the resulting microsome fractions were used as enzyme sources to evaluate the incorporation of C18:1 into *sn*-2 dominant C18:1 LPC. *sn*-2 dominant C18:1 LPC (20 μM) and oleoyl (18:1)-CoA (4 μM) were used as an acyl acceptor and an acyl donor, respectively. Data are shown as the mean ± SD of three replicates. Statistically significant differences (compared with negative control) are marked with asterisks indicating *P* values. ∗∗*P* < 0.01; ns indicates not significant; one-way ANOVA, Bonferroni’s multiple comparison test.

Because of the acyl migration reaction, lysoPLs are always a mixture of *sn*-1 and *sn*-2 isomers (21). In fact, the purity of both 1-hydroxy-2-C18:1-glycerophosphocholine (GPC) (*sn*-2 C18:1 LPC) and 1-C18:1-2-hydroxy-GPC (*sn*-1 C18:1 LPC), which we prepared and used as acyl acceptors in this study, was about 90%, contaminating about 10% of other isomers (Supplemental Fig. S1). Since *sn*-1 LPLAT activity incorporating C18:1 into *sn*-2 dominant C18:1-LPC was confirmed in HEK293A cells, these cells were used to screen for *sn*-1 LPLAT for C18:1 (Fig. 1). Expression of each of the 10 LPLATs (LPLAT5–14) was suppressed using siRNAs in HEK293A cells, and their membrane fractions were tested for *sn*-1 LPLAT activity incorporating C18:1 into *sn*-2 dominant LPC. The reduction of mRNA expression levels for each LPLAT was confirmed using qRT–PCR (Supplemental Fig S2). We found that the activity was significantly reduced in cells treated with siRNA for LPLAT10 (LPEAT2/LPCAT4) and slightly for LPLAT8 (LPCAT1) (Fig. 1). LPLAT8 was shown to incorporate FAs, mainly palmitic acid, at the *sn*-1 position besides *the sn*-2 position, thus it is an *sn*-1 LPLAT (22). These analyses suggested that LPLAT10 is a candidate for *sn*-1 LPLAT incorporating C18:1 at the *sn*-1 position of the glycerol backbone.

### In vitro substrate specificity

**Fig. 2:**
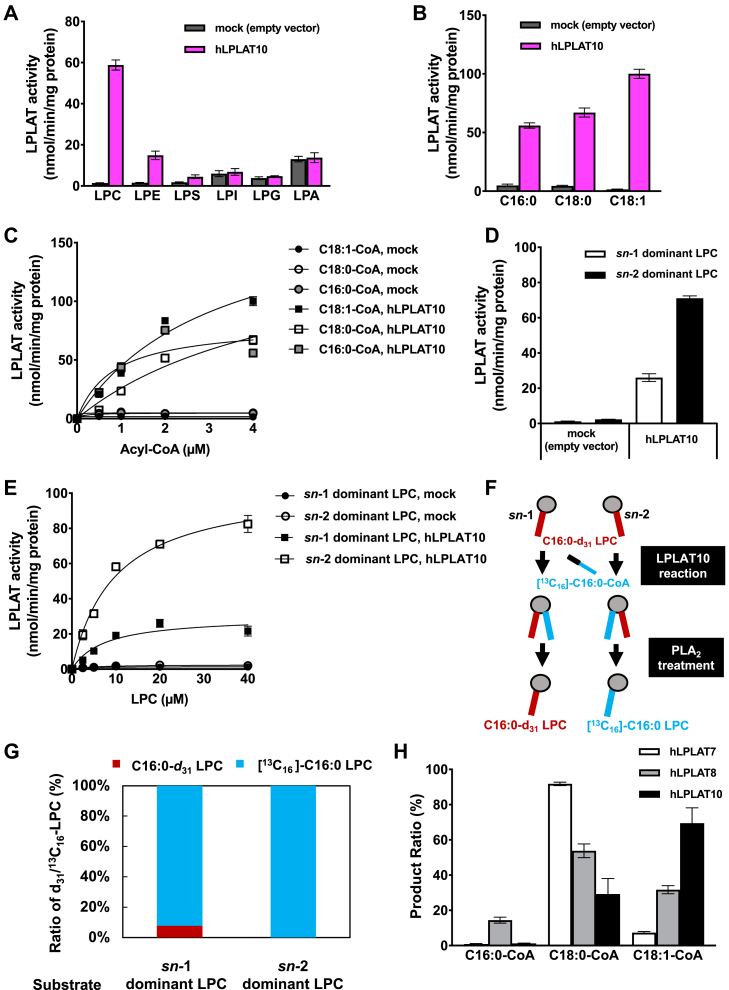
Biochemical characterization of LPLAT10. A: Polar head of acyl acceptor selectivity. Various lysoPLs with C18:1 at the *sn-*2 position dominant (20 μM) and C18:1-CoA (4 μM) were used as acyl acceptors and an acyl donor, respectively. Membrane fraction of HEK293A cells overexpressing human LPLAT10 was used for the enzyme source. Membrane fraction of HEK293A cells transfected with an empty vector was used as a control (mock). B and C: Acyl-CoA selectivity. LPC with C18:1 at the *sn-*2 position was dominant (20 μM) and various acyl-CoAs (C16:0-CoA or C18:0-CoA or C18:1-CoA, each 4 μM in (B) and varying concentration in (C)) were used as an acyl acceptor and acyl donor, respectively. Membrane fractions of HEK293A cells overexpressing human LPLAT10 were used for the enzyme source, and membrane fraction of HEK293A cells transfected with an empty vector was used as a control (mock). D and E: Positional selectivity (*sn*-1 or *sn*-2). LPC with C18:1 at the *sn*-1 or *sn*-2 position dominant (each 20 μM in (D) and varying concentration in (E)) were used as acyl acceptors, and C18:1-CoA (4 μM) was used as an acyl donor, respectively. Membrane fractions of HEK293A cells overexpressing human LPLAT10 were used as the enzyme source, and membrane fractions of HEK293A cells transfected with an empty vector were used as a control (mock). F and G: The ratio of two labeled FAs at the *sn*-1 position of the LPLAT10 reaction products. The reaction products were subjected to a PLA_2_ reaction. The amount of the resulting two *sn*-1 LPCs (1-[^13^C_16_] C16:0-2-hydroxy-GPC and 1-C16:0-*d*_31_-2-hydroxy-GPC) was determined by LC-MS/MS. H: Acyl-CoA competitive assay. LPC with C18:1 at the *sn-*2 position dominant (20 μM) and various acyl-CoA mixtures (C16:0-CoA or C18:0-CoA or C18:1-CoA, each 4 μM) were used as an acyl acceptor and acyl donor, respectively. Membrane fractions of HEK293A cells overexpressing human LPLAT10 were used as the enzyme source. Data in (A-E, H) are expressed as mean ± SD of triplicate measurements.

Substrate specificity of LPLAT10 was further examined in detail using a membrane fraction of HEK293A cells overexpressing human LPLAT10. First, we confirmed that the membrane fraction prepared from LPLAT10-overexpressing cells had 20-fold more activity to incorporate C18:1 into *sn*-2 dominant C18:1-LPC to generate diC18:1-PC than that from control cells (Fig. 2A). We also tested various *sn*-2 dominant lysoPLs and found that LPLAT10 used *sn*-2 dominant LPC, LPE, and LysoPS as acyl acceptors (Fig. 2A). Among the three lysoPLs, *sn*-2 dominant LPC was the best substrate. LPLAT10 did not act on *sn*-2 dominant LPI, LPG, and LPA (Fig. 2A). In the present condition, LPLAT10 was also found to utilize C16:0-CoA and C18:0-CoA as acyl donors in addition to C18:1-CoA (Fig. 2B). Of the three acyl-CoAs, C18:1-CoA was the better substrate, but C16:0-CoA and C18:0-CoA were also good substrates (Fig. 2B and C). We also determined the kinetic parameters (*K*_*m*_ and *V*_max_) for several acyl-CoAs and LPCs (Table 1), which showed that LPLAT10 had relatively higher activity toward unsaturated FA-CoA and *sn*-2 LPC.

**Table 1:** Kinetic parameters of LPLAT10

Substrate	*K*_*m*_ (μM) ± SEM	*V*_max_ (nmol/min/mg) ± SEM	*R*-squared
18:1-CoA	3.14 ± 0.75	186 ± 24	0.97
18:0-CoA	5.07 ± 1.72	157 ± 34	0.96
16:0-CoA	0.82 ± 0.36	81 ± 12	0.84
22:6-CoA	2.02 ± 0.78	4,297 ± 632	0.83
*sn*-1 dominant LPC	7.40 ± 2.09	29.8 ± 2.87	0.90
*sn*-2 dominant LPC	9.76 ± 1.01	105 ± 4.08	0.98

We then examined the position of the glycerol backbone into which LPLAT10 introduced an FA (*sn*-1 or *sn*-2) using 1-hydroxy-2-C18:1-GPC (*sn*-2 dominant C18:1 LPC) or 1-C18:1-2-hydroxy-GPC (*sn*-1 dominant C18:1 LPC) (90% purity each, Supplemental Fig. S1) as an acyl acceptor and C18:1-CoA as an acyl donor. Although LPLAT10 used both *sn*-1 and *sn*-2 C18:1 LPCs, the latter was found to be by far a better substrate (Fig. 2D and E). The enzymatic reaction was also performed using [^13^C_16_] C16:0-CoA as acyl donor and *sn*-2 dominant C16:0-*d*_31_-LPC (actually a mixture of 1-hydroxy-2-C16:0-*d*_31_-GPC and 1-C16:0-*d*_31_-2-hydroxy-GPC) as acyl acceptor, and the products, which were expected to be a mixture of 1-[^13^C_16_] C16:0-2-C16:0-*d*_31_-GPC and 1-C16:0-*d*_31_-2-[^13^C_16_] C16:0-GPC, were digested with PLA_2_ (Fig. 2F), and the resulting LPC was analyzed by LC-MS/MS. When *sn*-2 dominant C16:0-*d*_31_ LPC, which is composed mainly of 1-hydroxy-2-C16:0-*d*_31_-GPC (>90%), was used, the LPC detected was almost exclusively [^13^C_16_] C16:0 LPC (Fig. 2G), showing that LPLAT10 introduces an FA ([^13^C_16_] C16:0) at the *sn*-1 position of the C16:0-*d*_31_ LPC. [^13^C_16_] C16:0 LPC was detected dominantly even when *sn*-1 dominant C16:0-*d*_31_ LPC, which is composed mainly of 1-C16:0-*d*_*31*_-2-hydroxy-GPC (>90%), was used (Fig. 2G). These results clearly showed that LPLAT10 introduces an FA selectively at the *sn*-1 position of LPC.

To examine acyl-CoA selectivity, an acyl-CoA competition assay was performed for LPLAT7, LPLAT8, and LPLAT10. In this assay, the LPLAT reactions were performed under conditions where the same concentrations of the three acyl-CoAs (C16:0-, C18:0-, and C18:1-CoAs) were present. LPLAT10 preferentially utilized C18:1-CoA (Fig. 2H), whereas LPLAT7 and LPLAT8 mainly utilized C18:0-CoA and C16:0-CoA, respectively, in the same assay (Fig. 2H).

### PLs with unsaturated FAs at the *sn*-1 position are reduced in the brain of *Lplat10* KO mice

We generated *Lplat10* KO mice with a frameshift mutation in the third exon using a CRISPR-Cas9 system (Supplemental Fig. S3). *Lplat10* KO mice were born according to Mendel's laws and developed normally. *Lplat10* is known to be expressed highly in the mouse brain (30). We confirmed loss of LPLAT10 protein in the brain from KO mice by Western blot analysis (Supplemental Fig. S3B).

**Fig. 3:**
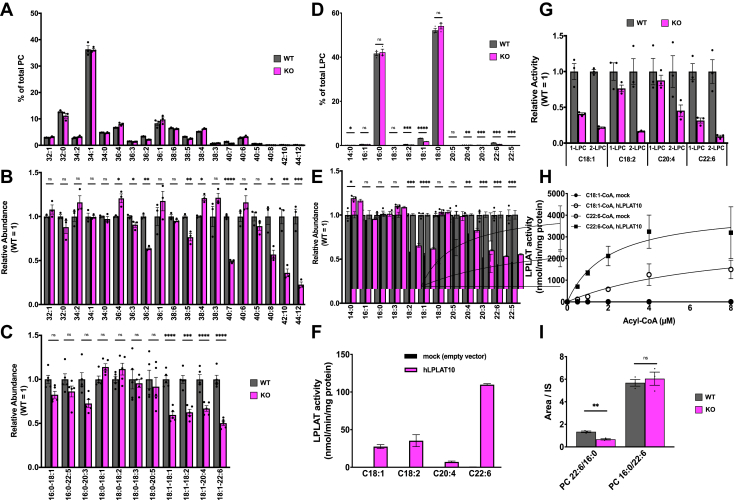
LPLAT10 determines the level of unsaturated FAs at the *sn*-1 position of PC in the mouse brain lipidomics analysis of the mouse brain from *Lplat10*-deficient mice. A-G and I: Gray and magenta bars represent wild-type and *Lplat10* KO mice, respectively. A and B: The abundance of PC species determined by LC-MS/MS positive ion mode. A: The ratio of each PC species. B: The abundance of each PC species in KO mice relative to wild type. Wild-type levels for each PC species are set to 1. C: Acyl chain composition of PC species determined by LC-MS/MS negative ion mode. Shown are the abundances of each PC species in KO mice relative to wild type. Wild-type levels for each PC species are set to 1. D and E: The abundance of LPC species prepared from phospholipids extracted from the mice brains treated with recombinant PLA_2_. D: The ratio of each LPC species. E: The abundance of each PC species in KO mice relative to wild type. Wild-type levels for each LPC species are set to 1. F–H: LPLAT10 activity using various unsaturated acyl-CoAs. LPC with C18:1 at the *sn-*2 dominant position (20 μM) and various acyl-CoAs (4 μM for (F) and (G), and varying concentrations in (H)) were used as an acyl acceptor and acyl donor, respectively. F and H: Membrane fraction of HEK293A cells transfected with an empty vector or human LPLAT10 was used for the enzyme source. G: Membrane fractions of the mice brains from wild-type or KO mice were used for the enzyme source. I: The abundance of positional isomers (C22:6/C16:0 PC, C16:0/C22:6 PC) in mice brain as determined by LC-MS/MS. All data are expressed as mean ± SD of triplicate measurements. ∗*P* < 0.05; ∗∗*P* < 0.01; ∗∗∗*P* < 0.001; ∗∗∗∗*P* < 0.0001; ns, not significant (*P* > 0.05) by two-tailed unpaired Student’s *t*-test.

Lipidomic analyses of the cerebrum (LC-MS/MS positive mode) revealed that noticeable changes were not observed for major PL species (Fig. 3A) but that certain minor PC species were reduced explicitly in *Lplat10* KO mice. For example, minor PC species, such as C36:2, C36:3, C38:5, C40:7, C40:8, C42:10, and C44:12 PC, were significantly reduced in *Lplat10* KO mice (Fig. 3B). LC-MS/MS analysis using negative mode assigned these PC species as C18:1_C18:1 PC for C36:2 PC, C18:1_C18:2 PC for C36:3 PC, C18:1_C20:4 PC for C38:5 PC, and C18:1_C22:6 PC for C40:7 PC, all of which potentially contain unsaturated FAs including C18:1 (Fig. 3C). Although we could not assign C40:8, C42:10, and C44:12 PC because of their lower abundance, they appeared to be C20:4_C20:4 PC, C20:4_C22:6 PC, and C22:6_C22:6 PC, respectively. Conversely, PC species, which potentially have saturated FAs, such as palmitic acid (C36:4 PC) or stearic acid (C38:4 PC) at the *sn*-1 position, were instead elevated (Fig. 3B). Among PE species, C40:8, C42:10, and C44:12 PE, which are thought to have two PUFAs, were significantly decreased (Supplemental Fig. S4). These results suggest that LPLAT10 contributes to the incorporation of unsaturated FAs at the *sn*-1 position of PC and PE in the mouse brain. However, it incorporated various FAs at the *sn*-1 position of PC in vitro (Fig. 2).

To detect FAs bound to the *sn*-1 position of PLs more directly, cerebrum PLs were enzymatically digested with PLA_2_, and the resulting lysoPLs were analyzed by LC-MS/MS. The analysis revealed that LPCs with unsaturated FAs, including C18:1, C18:2, C20:3, C20:4, 22:6, and C22:5 (docosapentaenoic acid) at the*sn*-1 position, were reduced explicitly in *Lplat10* KO mice (Fig. 3D and E). Of note, no differences were observed for major LPC species with C16:0 and C18:0 at the *sn*-1 position (Fig. 3D and E). These lipidomic analyses strongly indicated that LPLAT10 can incorporate various unsaturated FAs, including C18:1, C18:2, C20:3, C20:4, C22:6, and C22:5, at the *sn*-1 position.

### LPLAT10 incorporates various PUFAs at the *sn*-1 position

We re-examined the FA substrate specificity of LPLAT10, including PUFAs. As a result, LPLAT10 was found to show the activity to incorporate not only C18:1 but also PUFAs, such as C18:2, C20:4, and C22:6, at the *sn*-1 position, with the highest activity toward C22:6 (Fig. 3F). Furthermore, we examined whether the activity of LPLAT10 toward PUFAs also works in vivo by measuring the LPLAT10 activity using membrane fractions from the mouse brain. As a result, LPLAT activity that incorporated C18:1, C18:2, C20:4, and C22:6 into the *sn*-1 position of PC was detected in the mouse brain, and the LPLAT activity was significantly attenuated in the brains of *Lplat10* KO mice (Fig. 3G). We also examined whether C18:1-CoA or C22:6-CoA serves as a better substrate for LPLAT10 (Fig. 3H). The results revealed that LPLAT10 utilized both, but C22:6-CoA was found to be a better substrate. Consequently, we conducted a detailed analysis of the C38:6 PC species containing C22:6. Based on the method of Nakanishi *et al*. (2), we could separate two C38:6 PC isomers, 1-C16:0-2-C22:6-GPC and 1-C22:6-2-C16:0-GPC. In the brain from *Lplat10* KO mice, 1-C22:6-2-C16:0-GPC was significantly reduced (Fig. 3I), demonstrating that LPLAT10 contributes to the production of atypical PC species with C22:6 in the *sn*-1 position.

### *Lplat10* is highly expressed in neurons

**Fig. 4:**
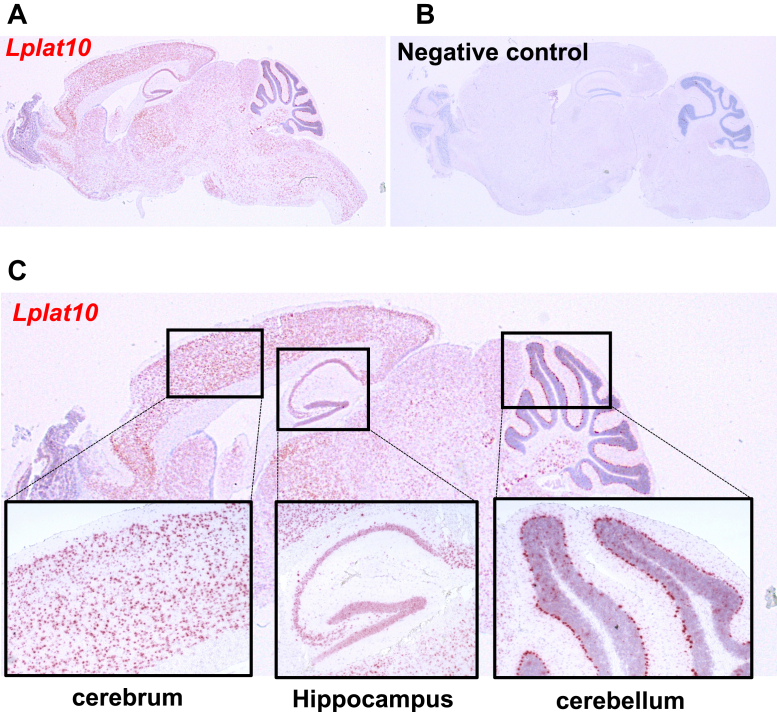
Expression of *Lplat10* in the mice brain. Detection of *Lplat10* mRNA in the mouse brain by in situ hybridization. Hybridization signals appear as red dots, indicating *Lplat10* mRNA localization. A and B: Low magnification view of the whole brain section hybridized with the antisense probe (A) and negative control probe (B). C: High magnification images of selected brain regions: cerebral cortex, hippocampus, and cerebellum.

**Fig. 5:**
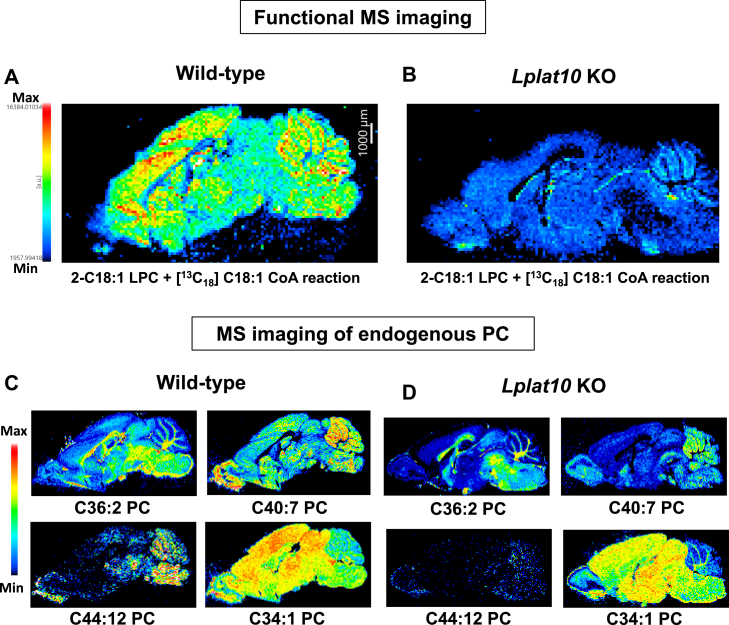
Visualization of LPLAT10 activity in the mouse brain. A and B: The distribution of LPLAT10 activity using [^13^C_18_] C18:1-CoA and *sn-*2 dominant C18:1 LPC at the brain sections from wild-type (A) and *Lplat10* KO (B) mice visualized using an fMSI technique. C and D: The distribution of endogenous PC species possibly containing oleic acid (C36:2, C34:1, and C40:7 PC) and DHA (C40:7 and C44:12 PC) in wild-type (C) and *Lplat10* KO (D) mice brain. The spatial resolution was 120 μm. The scale bars represent 1,000 μm.

We also performed in situ hybridization to examine the spatial localization of *Lplat*10 (Fig. 4A and B). *Lplat10* was found to be highly expressed in the cerebral cortex, hippocampus, and cerebellum, where it is expected to be expressed predominantly by neurons (Purkinje cells in the case of the cerebellum) (Fig. 4C). LPLAT10 enzyme activity was detected on the brain tissue section using an fMSI technique (Fig. 5). Unfixed wild-type mouse brain sections were loaded with the LPLAT10 substrates, *sn*-2 dominant C18:1 LPC (acyl acceptor) and [^13^C_18_] 18:1 CoA (acyl donor), and incubated. MSI detected the product, dioleoyl PC (C18:1_[^13^C_18_]C18:1 PC), in a wide area of the brain, with particularly high levels observed in the cerebral cortex, hippocampus, and cerebellum, where *Lplat*10 was highly expressed (Fig. 5A). On the other hand, dioleoyl PC (C18:1_[^13^C_18_]C18:1 PC) was barely detected in a section from *Lplat10* KO mouse brain (Fig. 5B). We also confirmed that endogenous PC species containing unsaturated FAs, which were decreased in *Lplat10* KO mice by LC-MS/MS analysis, were reduced in various brain regions (Fig. 5C and D).

## Discussion

LPLAT10, also known by several names, such as LPEAT2, LPCAT4, AYTL3, and AGPAT7, was identified by Cao *et al*. (30) in 2008 as an enzyme with notable activity in incorporating a variety of FAs—including C16:0, C18:0, C18:1, and C20:4—into ethanolamine-containing lysoPLs, that is, LPE. More recently, Eto *et al*. re-examined the enzymatic activity of LPLAT10/LPEAT2, revealing that it incorporated 22:6 (DHA) into LPE and LPC (31). These studies demonstrated the LPLAT activity of LPLAT10/LPEAT2 but did not specify the glycerol backbone positions for FA incorporation. They did not perform validation using gene-deficient cells or mice. In this study, we reconfirmed the LPLAT activity of LPLAT10/LPEAT2. Consistent with previous reports, our in vitro analysis showed that LPLAT10 utilized both LPC and LPE as substrates, with LPC being the preferred substrate (Fig. 2A). Considering its polar head group selectivity, LPLAT10 may utilize not only LPC and LPE as its substrates but also lysoPLs with dimethylethanolamine or monomethylethanolamine. We determined the precise substrate specificity and found that LPLAT10 had a selectivity for *sn*-2 lysoPLs with an FA at the *sn*-2 position (Fig. 2). Furthermore, by verifying the products of LPLAT10/LPEAT2 using KO mice (Fig. 3), we concluded that LPLAT10 is a novel *sn*-1 selective LPLAT that preferentially incorporates unsaturated FAs.

The spontaneous acyl chain migration from the *sn*-2 to *sn*-1 position within an LPL molecule has historically complicated the preparation of high-purity *sn*-2 LPLs required for *sn*-1 LPLAT assays (20, 22). Commercially available *sn*-2 LPLs are typically mixtures of *sn*-1 and *sn*-2 types, which complicates the precise determination of FA incorporation positions (21). In this study, we overcame this by preparing high-purity *sn*-2 LPLs under mildly acidic conditions and using them as acyl acceptors (22). In addition, to distinguish endogenous lipids from lipids produced by enzymatic reactions and to elucidate the specific incorporation sites on the glycerol backbone, we utilized deuterated LPLs acyl-CoAs and ^13^C-labeled acyl-CoAs (Fig. 2). The present study revealed several unique characteristics of LPLAT10: LPLAT10 is an *sn*-1 LPLAT with exclusive activity to incorporate an FA at the *sn*-1 position (Fig. 2D and E), a preference for LPC over LPE and LysoPS (Fig. 2A), and a notable affinity for DHA (Fig. 3F and H), the latter two findings aligning with Eto *et al*.’s ones (31).

It is often the case with some enzymes that their substrates and products diverge in vitro and in vivo. Lipidomics of the mouse brains revealed that only PLs with unsaturated FAs showed a slight but significant reduction in *Lplat10* KO mice (Fig. 3A–C), although LPLAT10 showed an activity to incorporate not only unsaturated FAs but also saturated FAs at the *sn*-1 position in vitro (Fig. 2). The reduction was more evident when analyzing LPLs from post-PLA_2_ treatment of brain-derived PLs (Fig. 3D and E). Recent studies, including ours, have shown that LPLAT7/LPGAT1 and LPLAT8/LPCAT1 specifically introduce C18:0 and C16:0 at the *sn*-1 position, respectively (22, 23, 25, 26). These enzymes are widely expressed in tissues, including the brain, and exhibit higher activities for C18:0-CoAs and C16:0-CoAs than LPLAT10 (22, 32), explaining why *Lplat10* KO mice maintain the levels of C18:0 and C16:0 PLs. In addition, both LPLAT7 and LPLAT8 exhibit extremely low activity in incorporating unsaturated FAs at the *sn*-1 position (22, 32). Therefore, the incorporation of unsaturated FAs at the *sn*-1 position is primarily contributed by LPLAT10. Recently, it was proposed that acyl-CoA synthetase long-chain family member 4 (ACSL4), an enzyme producing arachidonyl-CoA, and LPLAT11/LPIAT1/MBOAT7 are located in close proximity within the cells, enabling ACSL4 to efficiently supply arachidonyl-CoA to LPLAT11/LPIAT1/MBOAT7 (33). LPLAT10 may also efficiently synthesize PLs with a PUFA at the *sn*-1 position by pairing with some specific ACSLs.

Interestingly, according to Hama *et al*., LPLAT10 is implicated in the production of PC containing long-chain saturated FAs at the *sn*-1 position, relevant to X-linked adrenoleukodystrophy pathology. In ABCD1-deficient cells, a model cell for X-linked adrenoleukodystrophy, LPC with C26:0 at the *sn*-1 position, a biomarker of the disease, was significantly elevated (34), suggesting LPLAT10’s role in incorporating C26:0 into this unique PC species. These findings underscore LPLAT10’s broad substrate specificity, in contrast to the narrower specificities of LPLAT7 and LPLAT8, which preferentially utilize C18:0 and C16:0, respectively. The activity of LPLAT10 in incorporating diverse FAs at the *sn*-1 position, including unsaturated ones and C26:0, highlights its unique contribution to lipid diversity.

The biological roles of LPLAT10 and its products, mainly PC species with an unsaturated FA at the *sn*-1 position, remain elusive. *Lplat10* KO mice, which are born according to Mendelian ratios, show no overt abnormalities from HE staining of the cerebellum and immunostaining of Purkinje cells (Supplemental Fig. S5). Interestingly, Kuge *et al*. (35) demonstrated that 1-oleoyl-2-palmitoyl-PC, a minor PC species, recognized by a specific monoclonal antibody, localizes to a special membrane domain in neuronal projections and may influence neurite extension. Such a minor PC species might be produced by LPLAT10 and have unique functions if it localizes to the plasma membrane, which constitutes only a small percentage of the total cell membrane.

LPLAT10 is an LPLAT belonging to the AGPAT family and is highly homologous to LPLAT8/LPCAT1 and LPLAT9/LPCAT2, both of which have been shown to possess LPLAT activities (6). In the database, these *Lplat10* homologs are found to be present in species beyond fish but not in bacteria, plants, or archaea (Supplemental Fig. S6A). Interestingly, while *Lplat8* and *Lplat9* are conserved across all fish species, *Lplat10* is absent in *Chondrichthyes* but present in *Osteichthyes* among fish species and all species beyond fish (Supplemental Fig. S6B). This suggests that *Lplat10* evolved from *Lplat8* or *Lplat9* in *Osteichthyes*, probably by gene duplication during the evolution from *Chondrichthyes* to *Osteichthyes*. Future lipidomics studies in these fish species may uncover further insights into the functions of LPLAT10 and the PL species it produces.

## Data availability

All data are available upon request to the corresponding author.

## Supplemental data

This article contains supplemental data.

## Conflict of interest

The authors declare that they have no conflicts of interest with the contents of this article.

## Supplemental data

Supplemental Figure s
