# Supplementary material for: LPLAT10/LPEAT2 produces atypical phospholipids with an unsaturated FA at the sn-1 position
Source: J Lipid Res. 2026 Feb 23;67(4):101007. doi: 10.1016/j.jlr.2026.101007 (PMC13051972; doi:10.1016/j.jlr.2026.101007)
Supplement: Supplemental Figure — s [file mmc1.pptx]

## Slide 1
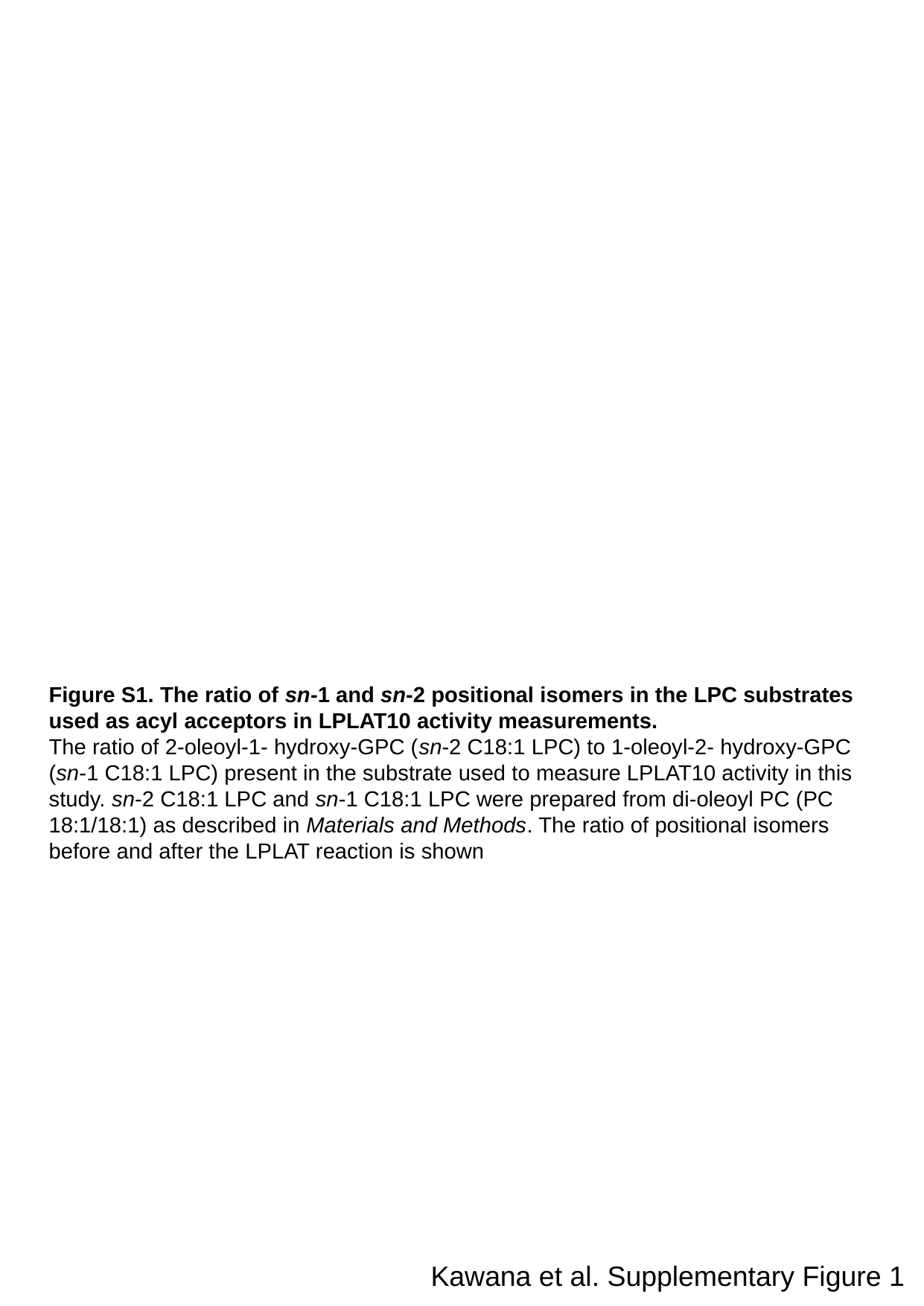

Figure S1. The ratio of sn-1 and sn-2 positional isomers in the LPC substrates used as acyl acceptors in LPLAT10 activity measurements.
The ratio of 2-oleoyl-1- hydroxy-GPC (sn-2 C18:1 LPC) to 1-oleoyl-2- hydroxy-GPC (sn-1 C18:1 LPC) present in the substrate used to measure LPLAT10 activity in this study. sn-2 C18:1 LPC and sn-1 C18:1 LPC were prepared from di-oleoyl PC (PC 18:1/18:1) as described in Materials and Methods. The ratio of positional isomers before and after the LPLAT reaction is shown
Kawana et al. Supplementary Figure 1

## Slide 2
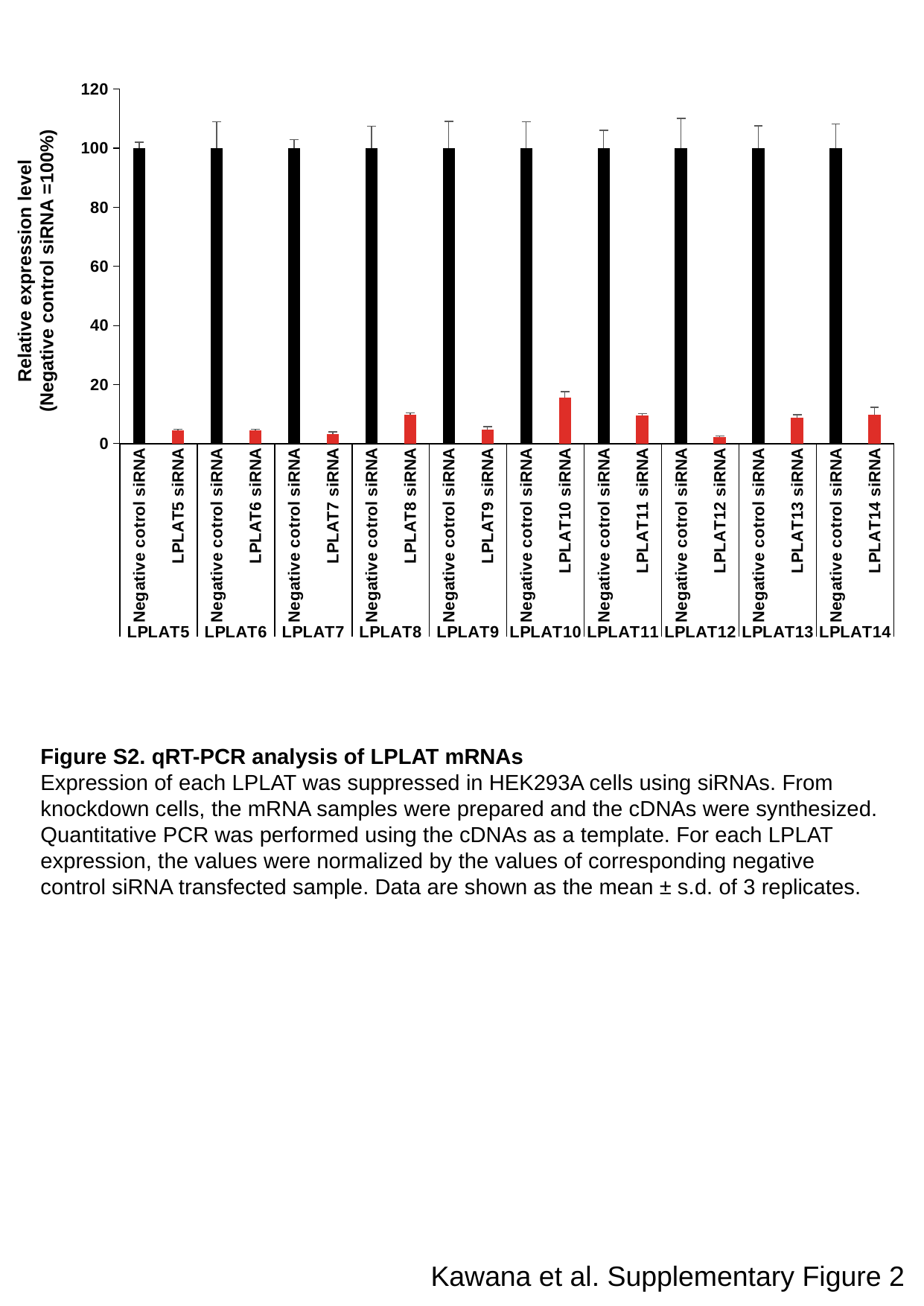

### Chart
| Category | Average |
|---|---|
| Negative cotrol siRNA | 100.0 |
| LPLAT5 siRNA | 4.477287838240925 |
| Negative cotrol siRNA | 100.0 |
| LPLAT6 siRNA | 4.428246269880086 |
| Negative cotrol siRNA | 100.0 |
| LPLAT7 siRNA | 3.2322328493386836 |
| Negative cotrol siRNA | 100.0 |
| LPLAT8 siRNA | 9.868665559880338 |
| Negative cotrol siRNA | 100.0 |
| LPLAT9 siRNA | 4.767573975046203 |
| Negative cotrol siRNA | 100.0 |
| LPLAT10 siRNA | 15.500284064064058 |
| Negative cotrol siRNA | 100.0 |
| LPLAT11 siRNA | 9.388263303932831 |
| Negative cotrol siRNA | 100.0 |
| LPLAT12 siRNA | 2.3067628712538766 |
| Negative cotrol siRNA | 100.0 |
| LPLAT13 siRNA | 8.770524261967891 |
| Negative cotrol siRNA | 100.0 |
| LPLAT14 siRNA | 9.841326266397305 |Relative expression level
(Negative control siRNA =100%)
Figure S2. qRT-PCR analysis of LPLAT mRNAs
Expression of each LPLAT was suppressed in HEK293A cells using siRNAs. From knockdown cells, the mRNA samples were prepared and the cDNAs were synthesized. Quantitative PCR was performed using the cDNAs as a template. For each LPLAT expression, the values were normalized by the values of corresponding negative control siRNA transfected sample. Data are shown as the mean ± s.d. of 3 replicates.
Kawana et al. Supplementary Figure 2

## Slide 3
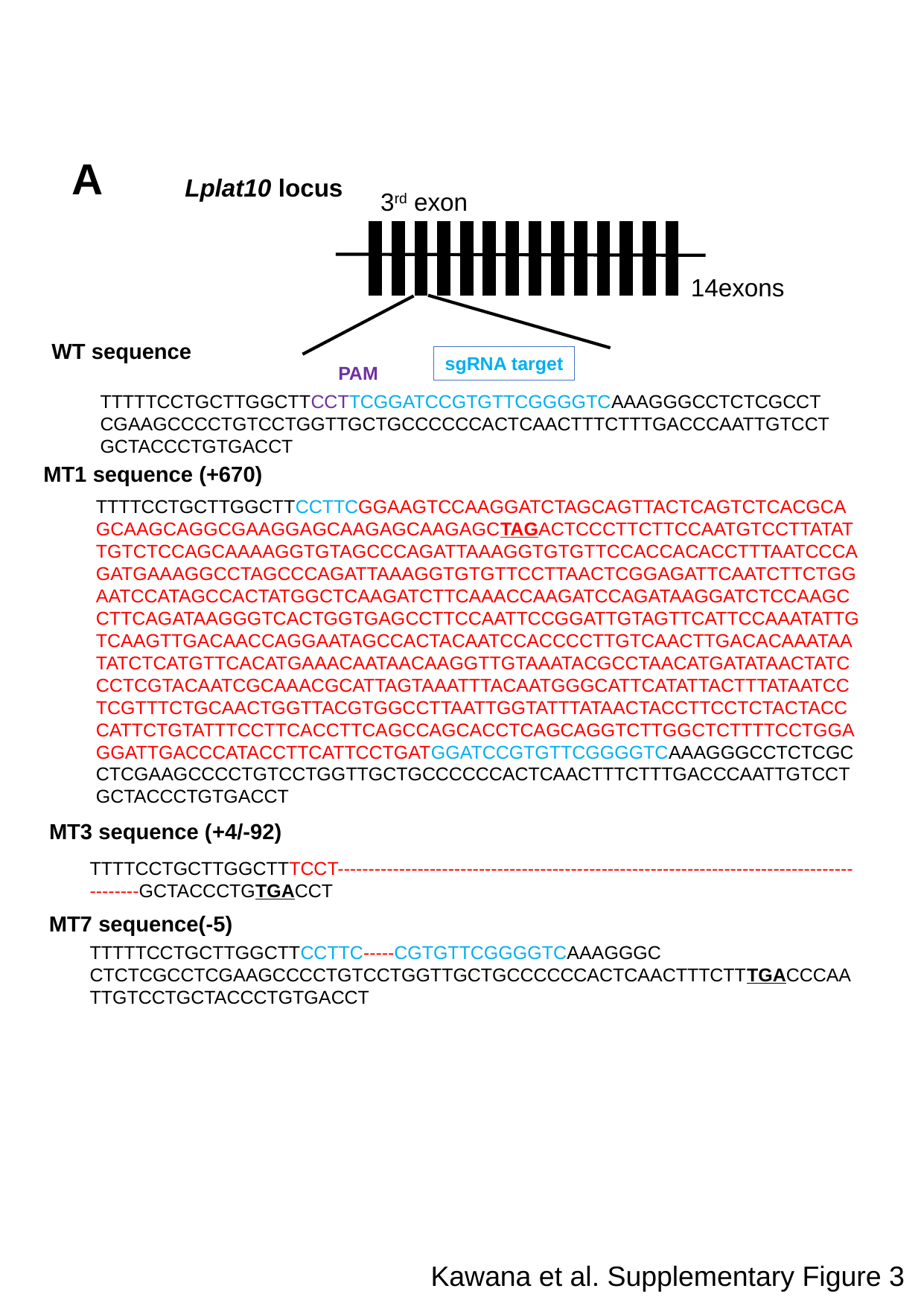

A
Lplat10 locus
3rd exon
14exons
WT sequence
sgRNA target
PAM
TTTTTCCTGCTTGGCTTCCTTCGGATCCGTGTTCGGGGTCAAAGGGCCTCTCGCCTCGAAGCCCCTGTCCTGGTTGCTGCCCCCCACTCAACTTTCTTTGACCCAATTGTCCTGCTACCCTGTGACCT
MT1 sequence (+670)
TTTTCCTGCTTGGCTTCCTTCGGAAGTCCAAGGATCTAGCAGTTACTCAGTCTCACGCAGCAAGCAGGCGAAGGAGCAAGAGCAAGAGCTAGACTCCCTTCTTCCAATGTCCTTATATTGTCTCCAGCAAAAGGTGTAGCCCAGATTAAAGGTGTGTTCCACCACACCTTTAATCCCAGATGAAAGGCCTAGCCCAGATTAAAGGTGTGTTCCTTAACTCGGAGATTCAATCTTCTGGAATCCATAGCCACTATGGCTCAAGATCTTCAAACCAAGATCCAGATAAGGATCTCCAAGCCTTCAGATAAGGGTCACTGGTGAGCCTTCCAATTCCGGATTGTAGTTCATTCCAAATATTGTCAAGTTGACAACCAGGAATAGCCACTACAATCCACCCCTTGTCAACTTGACACAAATAATATCTCATGTTCACATGAAACAATAACAAGGTTGTAAATACGCCTAACATGATATAACTATCCCTCGTACAATCGCAAACGCATTAGTAAATTTACAATGGGCATTCATATTACTTTATAATCCTCGTTTCTGCAACTGGTTACGTGGCCTTAATTGGTATTTATAACTACCTTCCTCTACTACCCATTCTGTATTTCCTTCACCTTCAGCCAGCACCTCAGCAGGTCTTGGCTCTTTTCCTGGAGGATTGACCCATACCTTCATTCCTGATGGATCCGTGTTCGGGGTCAAAGGGCCTCTCGCCTCGAAGCCCCTGTCCTGGTTGCTGCCCCCCACTCAACTTTCTTTGACCCAATTGTCCTGCTACCCTGTGACCT
MT3 sequence (+4/-92)
TTTTCCTGCTTGGCTTTCCT--------------------------------------------------------------------------------------------GCTACCCTGTGACCT
MT7 sequence(-5)
TTTTTCCTGCTTGGCTTCCTTC-----CGTGTTCGGGGTCAAAGGGC CTCTCGCCTCGAAGCCCCTGTCCTGGTTGCTGCCCCCCACTCAACTTTCTTTGACCCAATTGTCCTGCTACCCTGTGACCT
Kawana et al. Supplementary Figure 3

## Slide 4
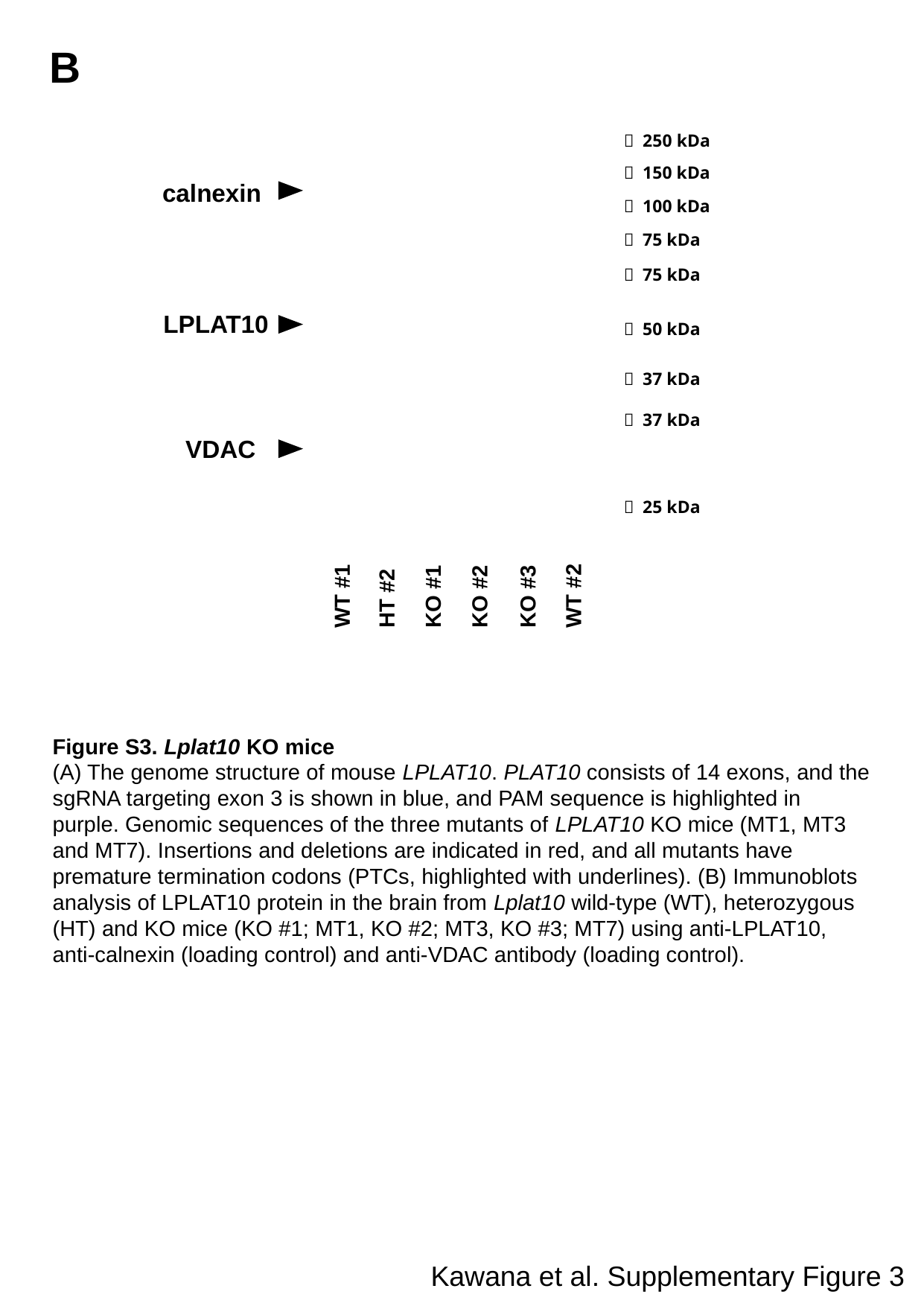

B
ー 250 kDa
ー 150 kDa
ー 100 kDa
ー 75 kDa
calnexin
ー 75 kDa
ー 50 kDa
ー 37 kDa
LPLAT10
ー 37 kDa
ー 25 kDa
VDAC
WT #1
HT #2
KO #1
KO #2
KO #3
WT #2
Figure S3. Lplat10 KO mice
(A) The genome structure of mouse LPLAT10. PLAT10 consists of 14 exons, and the sgRNA targeting exon 3 is shown in blue, and PAM sequence is highlighted in purple. Genomic sequences of the three mutants of LPLAT10 KO mice (MT1, MT3 and MT7). Insertions and deletions are indicated in red, and all mutants have premature termination codons (PTCs, highlighted with underlines). (B) Immunoblots analysis of LPLAT10 protein in the brain from Lplat10 wild-type (WT), heterozygous (HT) and KO mice (KO #1; MT1, KO #2; MT3, KO #3; MT7) using anti-LPLAT10, anti-calnexin (loading control) and anti-VDAC antibody (loading control).
Kawana et al. Supplementary Figure 3

## Slide 5
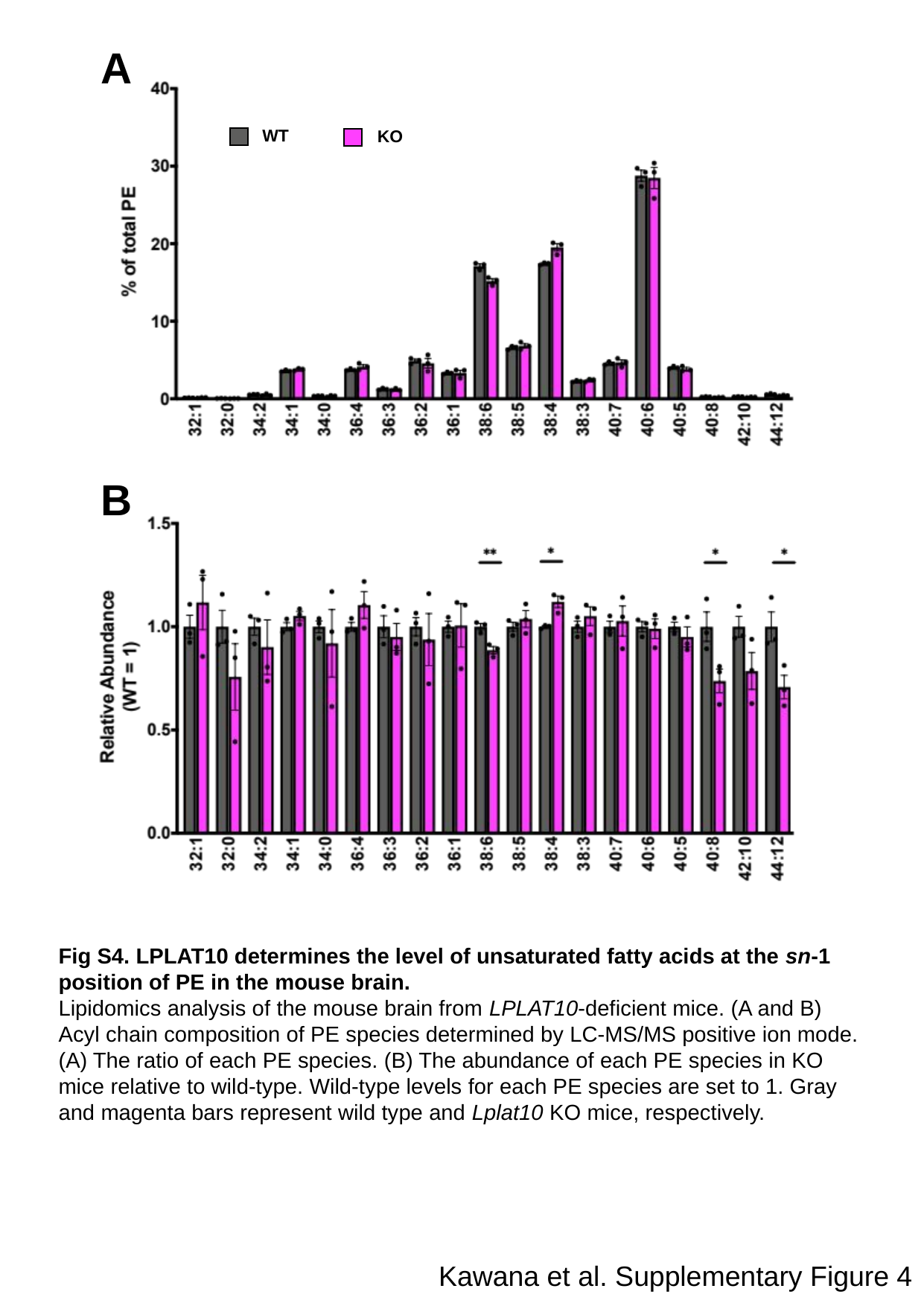

A
WT
KO
B
Fig S4. LPLAT10 determines the level of unsaturated fatty acids at the sn-1 position of PE in the mouse brain.
Lipidomics analysis of the mouse brain from LPLAT10-deficient mice. (A and B) Acyl chain composition of PE species determined by LC-MS/MS positive ion mode. (A) The ratio of each PE species. (B) The abundance of each PE species in KO mice relative to wild-type. Wild-type levels for each PE species are set to 1. Gray and magenta bars represent wild type and Lplat10 KO mice, respectively.
Kawana et al. Supplementary Figure 4

## Slide 6
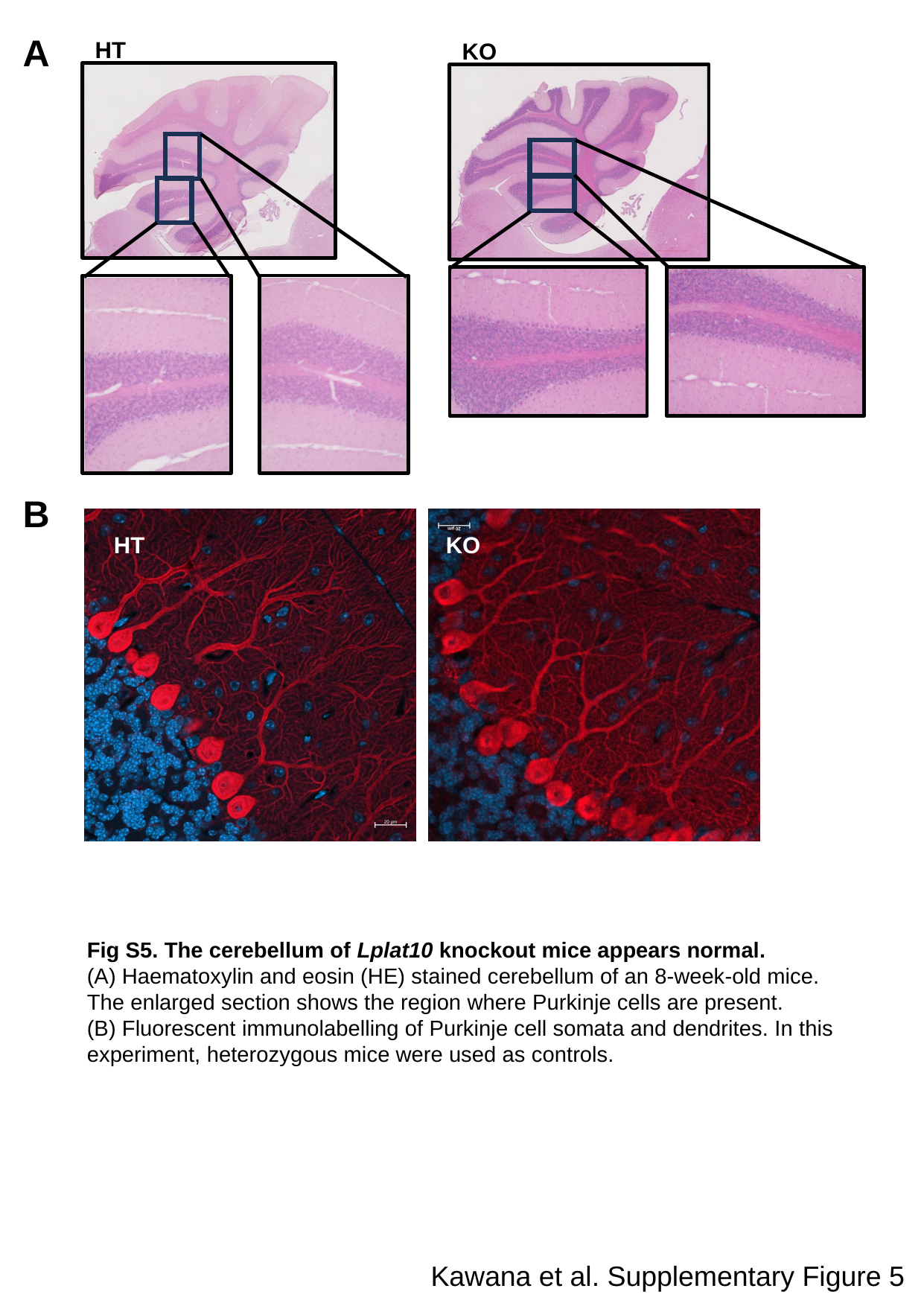

A
HT
KO
B
HT
KO
Fig S5. The cerebellum of Lplat10 knockout mice appears normal.
(A) Haematoxylin and eosin (HE) stained cerebellum of an 8-week-old mice. The enlarged section shows the region where Purkinje cells are present.
(B) Fluorescent immunolabelling of Purkinje cell somata and dendrites. In this experiment, heterozygous mice were used as controls.
Kawana et al. Supplementary Figure 5

## Slide 7
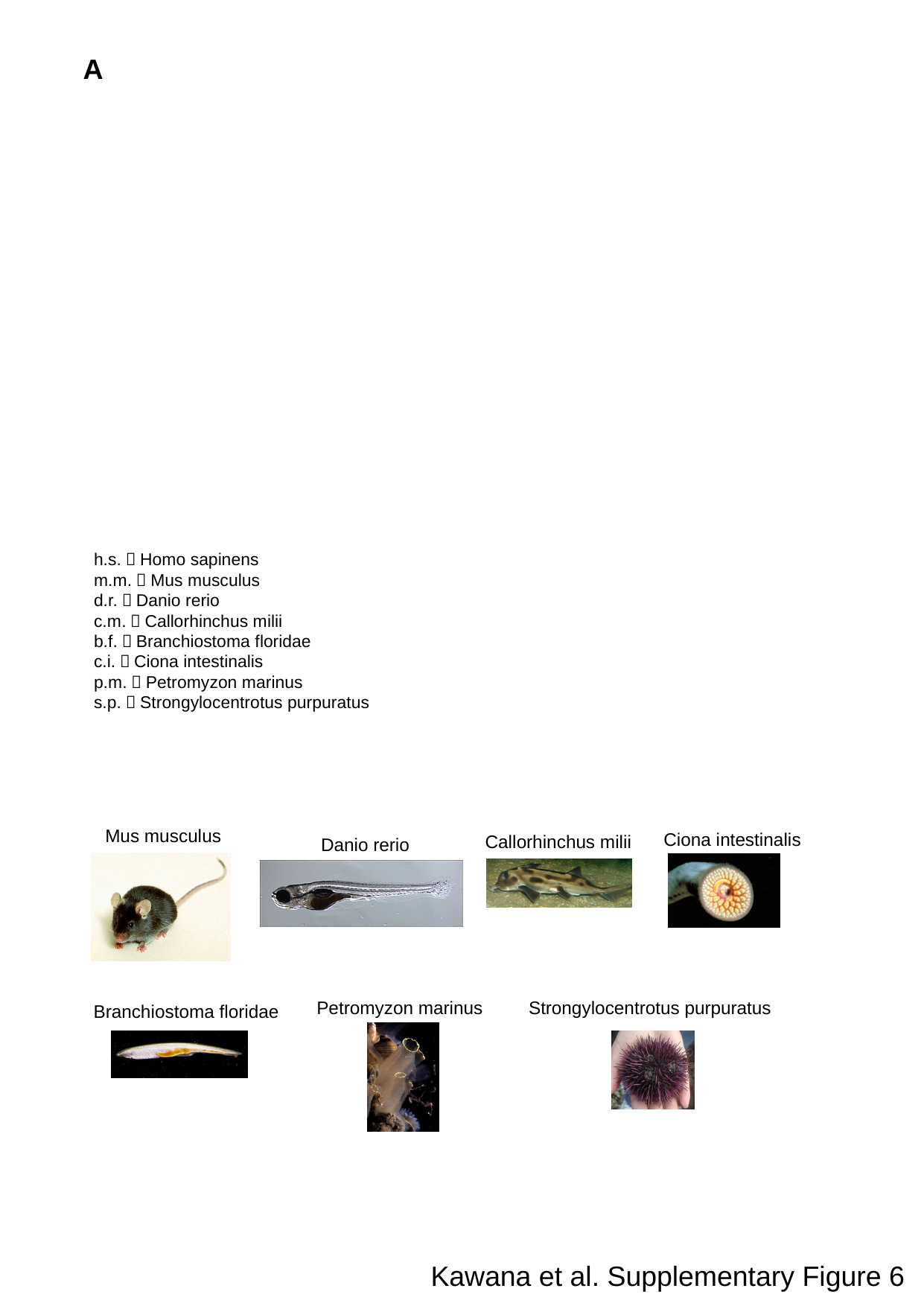

A
h.s.：Homo sapinens
m.m.：Mus musculus
d.r.：Danio rerio
c.m.：Callorhinchus milii
b.f.：Branchiostoma floridae
c.i.：Ciona intestinalis
p.m.：Petromyzon marinus
s.p.：Strongylocentrotus purpuratus
Mus musculus
Ciona intestinalis
Callorhinchus milii
Danio rerio
Petromyzon marinus
Strongylocentrotus purpuratus
Branchiostoma floridae
Kawana et al. Supplementary Figure 6

## Slide 8
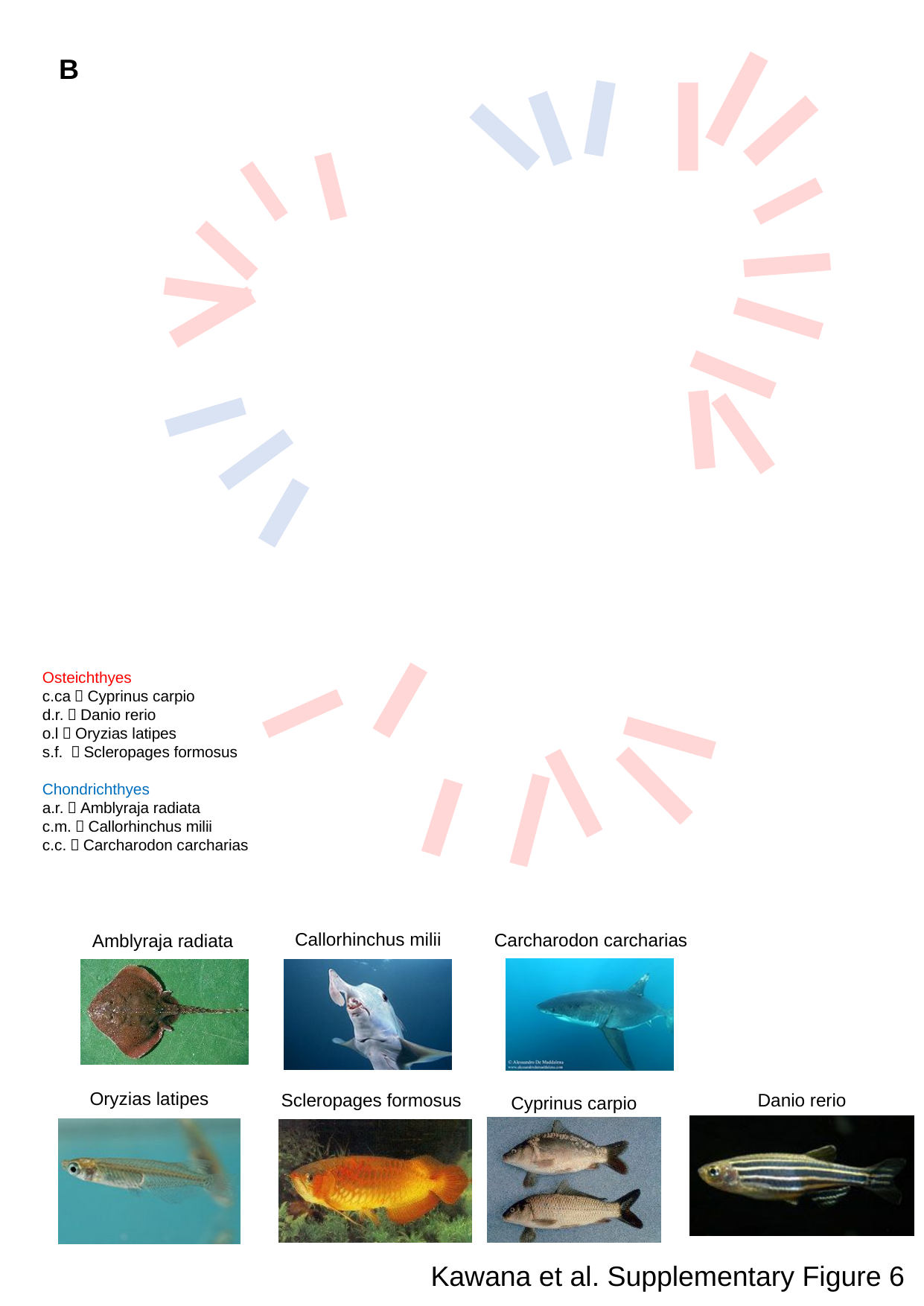

B
Osteichthyes
c.ca：Cyprinus carpio
d.r.：Danio rerio
o.l：Oryzias latipes
s.f. ：Scleropages formosus
Chondrichthyes
a.r.：Amblyraja radiata
c.m.：Callorhinchus milii
c.c.：Carcharodon carcharias
Callorhinchus milii
Carcharodon carcharias
Amblyraja radiata
Oryzias latipes
Scleropages formosus
Danio rerio
Cyprinus carpio
Kawana et al. Supplementary Figure 6

## Slide 9
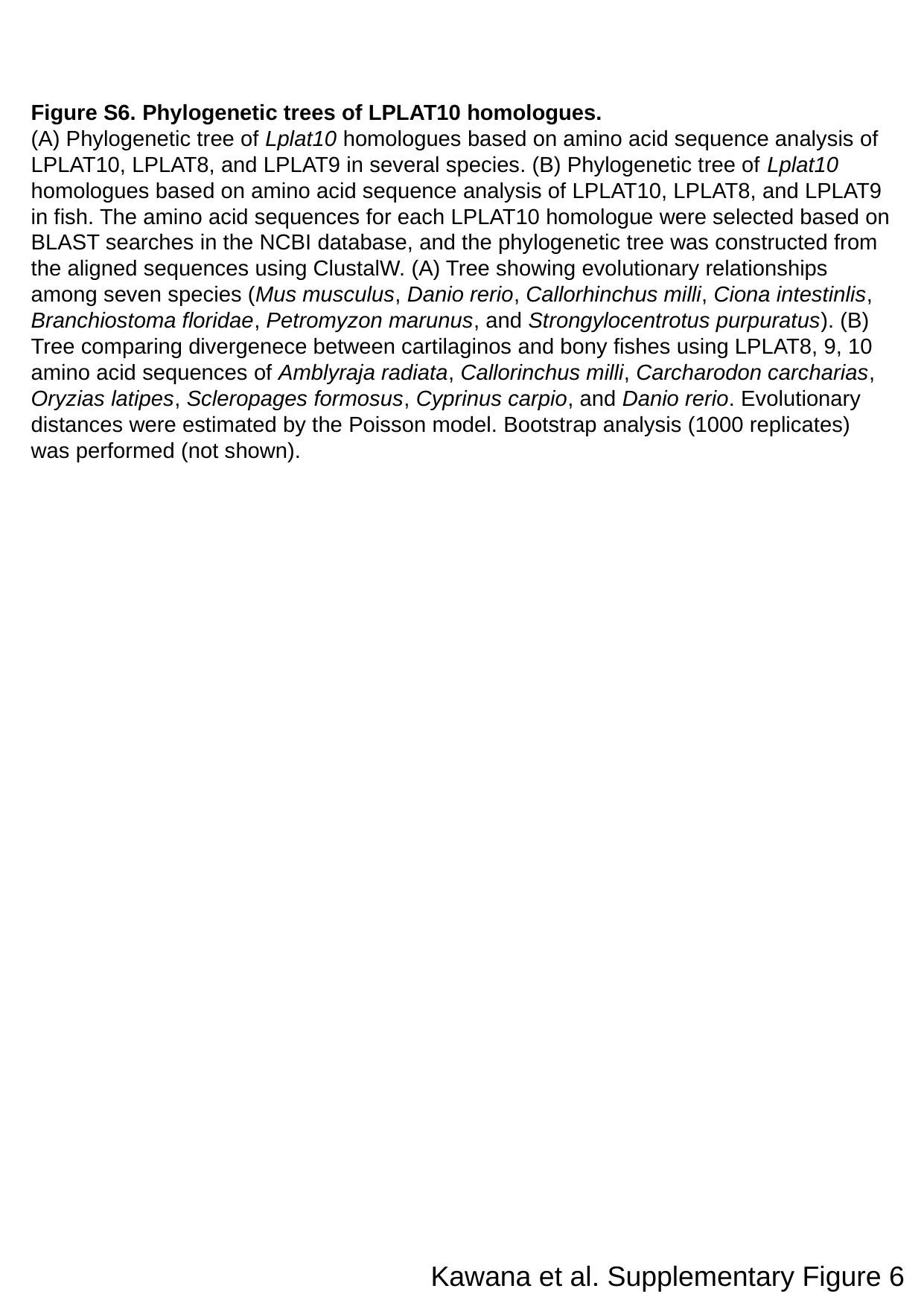

Figure S6. Phylogenetic trees of LPLAT10 homologues.
(A) Phylogenetic tree of Lplat10 homologues based on amino acid sequence analysis of LPLAT10, LPLAT8, and LPLAT9 in several species. (B) Phylogenetic tree of Lplat10 homologues based on amino acid sequence analysis of LPLAT10, LPLAT8, and LPLAT9 in fish. The amino acid sequences for each LPLAT10 homologue were selected based on BLAST searches in the NCBI database, and the phylogenetic tree was constructed from the aligned sequences using ClustalW. (A) Tree showing evolutionary relationships among seven species (Mus musculus, Danio rerio, Callorhinchus milli, Ciona intestinlis, Branchiostoma floridae, Petromyzon marunus, and Strongylocentrotus purpuratus). (B) Tree comparing divergenece between cartilaginos and bony fishes using LPLAT8, 9, 10 amino acid sequences of Amblyraja radiata, Callorinchus milli, Carcharodon carcharias, Oryzias latipes, Scleropages formosus, Cyprinus carpio, and Danio rerio. Evolutionary distances were estimated by the Poisson model. Bootstrap analysis (1000 replicates) was performed (not shown).
Kawana et al. Supplementary Figure 6
